# The Ccr4-Not complex regulates TORC1 signaling and mitochondrial metabolism by promoting vacuole V-ATPase activity

**DOI:** 10.1371/journal.pgen.1009046

**Published:** 2020-10-16

**Authors:** Hongfeng Chen, P. Winston Miller, Daniel L. Johnson, R. Nicholas Laribee

**Affiliations:** 1 Department of Pathology and Laboratory Medicine, College of Medicine and the Center for Cancer Research, University of Tennessee Health Science Center, Memphis, TN, United States of America; 2 Molecular Bioinformatics Core and the University of Tennessee Health Science Center Office of Research, University of Tennessee Health Science Center, Memphis, TN, United States of America; UC Davis, UNITED STATES

## Abstract

The Ccr4-Not complex functions as an effector of multiple signaling pathways that control gene transcription and mRNA turnover. Consequently, Ccr4-Not contributes to a diverse array of processes, which includes a significant role in cell metabolism. Yet a mechanistic understanding of how it contributes to metabolism is lacking. Herein, we provide evidence that Ccr4-Not activates nutrient signaling through the essential target of rapamycin complex 1 (TORC1) pathway. Ccr4-Not disruption reduces global TORC1 signaling, and it also upregulates expression of the cell wall integrity (CWI) pathway terminal kinase Mpk1. Although CWI signaling represses TORC1 signaling, we find that Ccr4-Not loss inhibits TORC1 independently of CWI activation. Instead, we demonstrate that Ccr4-Not promotes the function of the vacuole V-ATPase, which interacts with the Gtr1 GTPase-containing EGO complex to stimulate TORC1 in response to nutrient sufficiency. Bypassing the V-ATPase requirement in TORC1 activation using a constitutively active Gtr1 mutant fully restores TORC1 signaling in Ccr4-Not deficient cells. Transcriptome analysis and functional studies revealed that loss of the Ccr4 subunit activates the TORC1 repressed retrograde signaling pathway to upregulate mitochondrial activity. Blocking this mitochondrial upregulation in Ccr4-Not deficient cells further represses TORC1 signaling, and it causes synergistic deficiencies in mitochondrial-dependent metabolism. These data support a model whereby Ccr4-Not loss impairs V-ATPase dependent TORC1 activation that forces cells to enhance mitochondrial metabolism to sustain a minimal level of TORC1 signaling necessary for cell growth and proliferation. Therefore, Ccr4-Not plays an integral role in nutrient signaling and cell metabolism by promoting V-ATPase dependent TORC1 activation.

## Author summary

Eukaryotic cells dynamically control nutrient signaling pathways to regulate metabolism, anabolic growth, and cell proliferation through molecular effectors that remain poorly understood. The Ccr4-Not complex is a multiprotein regulatory factor conserved from yeast to humans that integrates both mRNA degradation and ubiquitin ligase activities. While budding yeast Ccr4-Not represses mitochondrial metabolism to allow normal glycolytic metabolism, how it does so is not clear. Herein, we demonstrate that Ccr4-Not activates nutrient signaling through the conserved target of rapamycin complex 1 (TORC1) pathway by promoting the function of the vacuolar V-ATPase proton pump that acidifies the vacuole and activates TORC1. Ccr4-Not deficient cells exhibit decreased V-ATPase stability, leading to defects in both vacuole acidification and TORC1 signaling. This TORC1 defect causes an adaptive upregulation of mitochondrial metabolism to maintain a minimal level of TORC1 activity. This study defines a previously unknown role for Ccr4-Not as a key activator of a conserved eukaryotic nutrient signaling pathway highly relevant to human health and disease.

## Introduction

Eukaryotic cells respond to nutrient flux by modifying signaling through pathways that promote adaptive growth and proliferative responses. These mechanisms allow cells to dynamically adjust their metabolism to coordinate growth and proliferation with the availability of the nutrients that support these processes [1]. This regulation involves coordinated control at the different stages of the gene expression pathway, including at the level of gene transcription, mRNA stability, protein translation, and protein degradation [2]. Oftentimes these regulators function at multiple steps in the gene expression process to ensure efficient and coordinated responses to environmental nutrient flux. Understanding how such shared regulators operate within the context of nutrient signaling is essential for determining how cells adapt to shifts in their nutrient environment.

The evolutionarily conserved Ccr4-Not complex regulates gene expression at the transcriptional and post-transcriptional stages, while it also contributes to translational control and proteostasis maintenance [3]. Its role in these diverse pathways highlights its wide-ranging importance in the cell, which is further underscored by the embryonic lethality caused by loss of individual Ccr4-Not members [4, 5]. Yet how Ccr4-Not mechanistically regulates these diverse activities remains unclear. Ccr4-Not is best characterized in budding yeast where it exists as an approximately 1 MDa complex containing the Ccr4, Caf1, Caf40, Caf130, and Not1-5 subunits [3]. The complex has a modular organization with the Ccr4 and Caf1, Caf40 and Caf130, and the Not2-5 subunits forming distinct sub-modules organized onto a Not1 scaffold [6]. Ccr4 is an mRNA deadenylase that degrades polyadenylated mRNA, and it functions as the main eukaryotic deadenylase [7, 8]. The Not4 subunit is a RING domain ubiquitin ligase that additionally has an RNA recognition motif (RRM) and C3H1 domain (referred to as the RRM-C domain), which are candidate RNA binding domains [9–11]. Whether Not4 binds RNA remains unknown, although the RRM-C domain contributes to Not4 *in vivo* ligase activity suggesting Ccr4-Not may integrate RNA metabolism with ubiquitin ligation [11]. The Not2, Not3, and Not5 subunits likely contribute regulatory and structural functions to the complex [3]. While the role of the yeast Caf40 and Caf130 submodule remains unknown, the *Drosophila* and mammalian Caf40 orthologs recruit Not4 ligases into the Ccr4-Not complex [12, 13].

Since Ccr4-Not controls every stage of the gene expression pathway, it is an ideal effector for integrating global responses to nutrient flux. Support for this connection has been provided by studies demonstrating that yeast Ccr4-Not acts within both the glucose-activated Ras/PKA, and the nutrient activated target of rapamycin complex 1 (TORC1), pathways [14–16]. Specifically, the Not3 and Not5 subunits exhibit post-translational modification differences in response to changes in Ras/PKA signaling, while Ccr4-Not mutants activate Ras/PKA repressed transcription factors [15]. Carbon source also controls Not1 protein expression since cells cultured in a non-fermentable carbon source reduce Not1 expression relative to fermentatively (glycolytic) growing cells [17]. While the impact this regulation has on the cell remains unclear, one predicted consequence is that decreased Ccr4-Not activity may contribute to the shift from glycolytic metabolism to mitochondrial-dependent oxidative phosphorylation upon glucose exhaustion. Consistent with this possibility, budding yeast and *Candida albicans* lacking the Ccr4 or Caf1 subunits upregulate mitochondrial metabolism in glucose-rich conditions [18–20]. Additionally, glucose exhaustion activates the Yak1 kinase, which phosphorylates Caf1 to promote the transient G1 arrest required for cells to undergo diauxic shift [21]. Ccr4 also binds nutrient-regulated transcripts to facilitate their degradation, thus further reinforcing the connections between Ccr4-Not and the regulation of cell metabolism at multiple stages of the gene expression pathway [22].

How Ccr4-Not contributes to nutrient signaling and cellular metabolism is best understood for the TORC1 (also known as the mechanistic TORC1) pathway. TORC1 is a highly conserved serine/threonine kinase complex that transmits nutrient (predominantly amino acid) information to the transcriptional and translational machinery promoting anabolism and cell proliferation [23]. Yeast chemical genetic screening identified Ccr4-Not mutants to be sensitive to the TORC1 inhibitor rapamycin, thus genetically connecting Ccr4-Not to TORC1 [14]. Subsequently, TORC1-dependent transcription of ribosomal RNA was shown to require Ccr4-Not [16]. Yet whether Ccr4-Not contributes to additional aspects of the TORC1 pathway has remained unclear. Since mammalian Ccr4-Not may be a component of the mTORC1 pathway [24, 25], determining how yeast Ccr4-Not impacts TORC1 signaling could clarify how mammalian Ccr4-Not affects mTORC1 signaling.

TORC1 consists of either the Tor1 or Tor2 kinases, the essential subunits Lst8 and Kog1, and the non-essential Tco89 subunit [26]. TORC1 constitutively localizes to the vacuole (yeast lysosome) membrane where it interacts with the EGO and V-ATPase complexes, which activate TORC1 in response to nutrient availability [27, 28]. EGO consists of the Ego1-3 subunits and the conserved Rag GTPases Gtr1 and Gtr2 [29]. The V-ATPase is a conserved multimeric H^+^ pump that acidifies the vacuole compartment to facilitate the degradation of macromolecules required for vacuole-dependent nutrient import, recycling, and storage [30]. In mammals, this process provides the free amino acids in the lysosomal lumen needed for mTORC1 activation [23, 31], yet whether yeast EGO and V-ATPase directly sense vacuole amino acids to activate TORC1 has yet to be demonstrated. However, V-ATPase interaction with EGO does have a critical role in TORC1 activation in response to nutrient (predominantly amino acids) sufficiency [28]. The V-ATPase consists of two (V_0_ and V_1_) sub-complexes such that V_0_ is embedded in the vacuole membrane, while V_1_ interacts with V_0_ on the cytoplasmic face. The V_1_ sub-complex contains an integral ATPase activity that, when bound to V_0_, hydrolyzes ATP to provide the energy for extracting cytoplasmic H^+^ and pumping them into the vacuole [30]. While mutations that disrupt EGO or the V-ATPase drastically impair TORC1 activity, they do not abolish it [27, 28]. Additional amino acid sensing mechanisms exist, including leucyl tRNA synthetase, which binds leucine and interacts with EGO to stimulate TORC1 [32]. Glutamine also activates TORC1 independently of EGO, although the glutamine source and how it mediates TORC1 activation remains unknown [33]. Activated TORC1 then signals through three downstream effector pathways to promote anabolism and cell proliferation. TORC1 stimulates phosphorylation of ribosomal protein S6 (S6ph) by direct phosphorylation of the kinase Ypk3, which stimulates translation [34, 35], and it directly phosphorylates the Sch9 kinase to activate growth-promoting transcriptional and translational pathways [36]. TORC1 also phosphorylates the regulatory factor Tap42 to inactivate PP2A and PP2A-like phosphatases to prevent dephosphorylation and activation of nutrient-stress responsive transcription factors such as Gln3 [37].

In this report, we demonstrate that Ccr4-Not deficiency dramatically impairs TORC1 signaling, and it also deregulates expression of the Mpk1/Slt2 (hereafter referred to as Mpk1) mitogen activated protein kinase (MAPK) kinase that is a downstream effector of the CWI stress signaling pathway that inhibits TORC1. However, we find that TORC1 inhibition in a Ccr4-Not mutant (*ccr4Δ*) is independent of Mpk1 deregulation or CWI signaling. Instead, we demonstrate that Ccr4-Not disruption reduces the stability and function of the vacuole V-ATPase, which causes vacuole acidification defects and reduced TORC1 signaling. Bypassing the V-ATPase requirement in TORC1 activation through expression of a constitutively active Gtr1 GTPase fully restores TORC1 activity in *ccr4Δ*. However, this Gtr1 mutant fails to restore growth of *ccr4Δ* under TORC1 inhibitory conditions, suggesting Ccr4-Not has additional effects on TORC1 signaling downstream of the V-ATPase. Transcriptome analysis (RNA-seq) and functional studies reveal that Ccr4-Not disruption increases mitochondrial metabolism, which TORC1 normally represses by inhibiting mitochondrial retrograde signaling. Intriguingly, preventing mitochondrial upregulation in *ccr4Δ* further impairs TORC1 signaling and leads to mitochondrial metabolic defects. These data suggest that the enhanced mitochondrial metabolism in Ccr4-Not mutants functions as an adaptive response required to sustain a minimal level of TORC1 signaling needed for cell growth and proliferation.

## Results

### Ccr4-Not activates TORC1 signaling independently of its role in mRNA deadenylation

**Fig 1:**
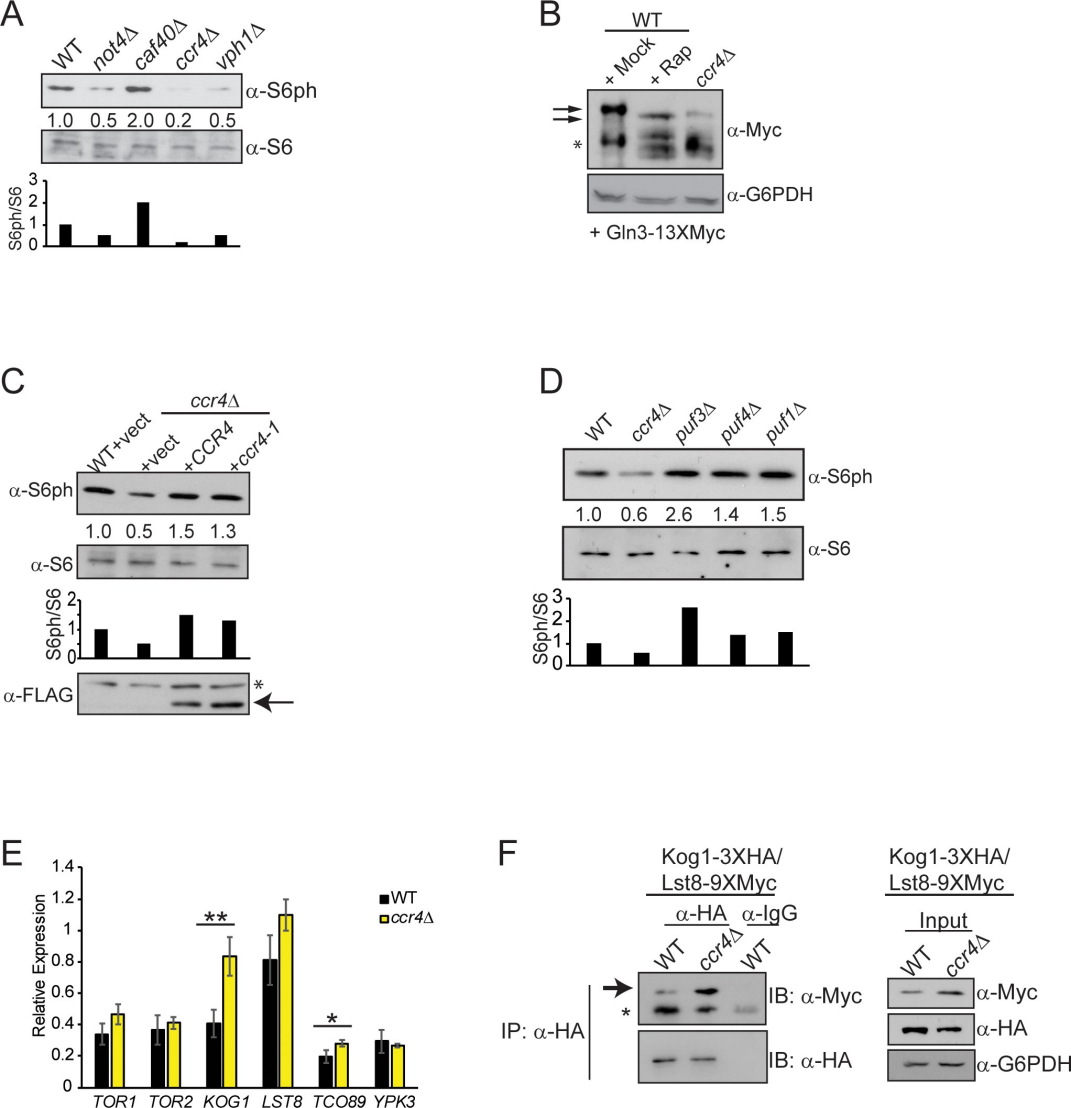
Ccr4-Not regulates TORC1 signaling. (A) TORC1 activity was analyzed by performing immunoblot (IB) analysis for S6ph and total S6 from WT, *not4Δ*, *caf40Δ*, *ccr4Δ*, and *vph1Δ* cell extracts. Numbers reflect the S6ph/S6 ratio with WT set to 1 and the mutant S6ph/S6 ratio normalized to WT. The data also are represented as a histogram, and they are representative of three independent experiments. (B) WT and *ccr4Δ* were transformed with a Gln3-13XMyc expression plasmid and then cultured in SC selective media to mid-log phase. The WT was then mock treated or treated with 200 nM rapamycin for 30 minutes before harvesting. Total cell extracts were resolved in an 8% SDS-PAGE gel containing 150 μM Phostag reagent and then probed by α-Myc IB to detect Gln3. The arrows indicate hyperphosphorylated (upper) and hypophosphorylated (lower) Gln3, while the asterisk indicates Gln3 cleavage products. Glucose-6-phosphate dehydrogenase (G6PDH) levels were analyzed as a loading control. The results are representative of three independent experiments. (C) Analysis of TORC1 in WT and *ccr4Δ* transformed with control vector, or vector expressing FLAG-tagged wild-type Ccr4 or the enzymatically inactive ccr4-1 mutant. Samples were analyzed and quantified as described in (A), and the results are representative of four independent experiments. (D) Analysis of TORC1 signaling in WT, *ccr4Δ*, and various PUF deletion mutants performed as in (A). Data are representative of three independent experiments. (E) RT-qPCR analysis of the indicated TORC1 pathway genes normalized to the *SPT15* housekeeping gene. Data are the average and standard deviation (SD) of three independent experiments with pairwise Student’s t-test performed to test significance. *- *p<0*.*05*; **- *p<0*.*01*. (F) WT and *ccr4Δ* expressing Kog1-6XHA and Lst8-9XMyc were cultured to mid-log phase before harvesting and performing the indicated immunoprecipitations (IPs) and IBs. The arrow indicates the Lst8-specific band and the asterisk denotes the IgG heavy chain. The data are representative of three independent experiments.

To further define how Ccr4-Not functions in the TORC1 pathway, we cultured wild-type (WT) cells, as well as cells lacking the Not4 ubiquitin ligase (*not4Δ*), the Caf40 subunit (*caf40Δ*), or the Ccr4 mRNA deadenylase (*ccr4Δ*) to mid-log phase in nutrient rich (YPD) media. As a control for impaired TORC1 signaling, we analyzed a mutant lacking the vacuole-specific V-ATPase subunit Vph1 (*vph1Δ*) [28]. As expected, *vph1Δ* reduced TORC1 activity while both *ccr4Δ* and *not4Δ*, but not *caf40Δ*, also decreased TORC1 signaling, which was determined by quantifying S6ph (Fig 1A). Because *not4Δ* grows poorly, we analyzed the *ccr4Δ* further to define how Ccr4-Not activates TORC1 since *ccr4Δ* has only a minor growth phenotype. To confirm the TORC1 signaling defect using an independent readout, WT and *ccr4Δ* were transformed with a plasmid expressing Myc-tagged Gln3. Gln3 is phosphorylated when TORC1 is active, and it becomes rapidly dephosphorylated upon TORC1 inhibition [37, 38]. Cells were grown to mid-log phase, and Gln3 phosphorylation was detected by mobility shift assay using Phostag impregnated SDS-PAGE, which separates proteins based on their phosphorylation state [39, 40]. As a control for TORC1 inhibition, WT cells were treated with 200 nM rapamycin (Rap) for 30 minutes before harvesting. Hyperphosphorylated Gln3 (indicated by upper arrow in Fig 1B) was detected in mock-treated WT cells, while rapamycin-mediated TORC1 inhibition increased Gln3 mobility thus indicating decreased Gln3 phosphorylation (denoted by the bottom arrow in Fig 1B). Gln3 mobility from *ccr4Δ* resembled that from the rapamycin-treated WT cells, thus indicating *ccr4Δ* has reduced TORC1 signaling (Fig 1B). Therefore, two independent readouts for TORC1 activity indicate that Ccr4-Not disruption decreases signaling through this pathway.

To determine if Ccr4-Not activates TORC1 via its role in mRNA degradation, WT and *ccr4Δ* were transformed with either control vector or, in *ccr4Δ*, with vector overexpressing either FLAG-tagged *CCR4* or the catalytically inactive *ccr4-1* mutant [8, 16]. Cells were grown in nutrient defined synthetic complete (SC) media lacking uracil (to select for plasmid maintenance), and then they were analyzed for TORC1 activity. The *ccr4Δ* vector control cells exhibited reduced S6ph relative to WT, while both *CCR4* and *ccr4-1* restored S6ph levels (Fig 1C). These data indicate Ccr4-Not activates TORC1 independently of its role in mRNA degradation. To provide further support that the TORC1 signaling defect is independent of Ccr4-Not mediated mRNA decay, we analyzed TORC1 in cells lacking individual Pumilio (PUF) family RNA binding proteins, including Puf3 and Puf4, which recruit Ccr4-Not to many of its mRNA substrates for deadenylation [22, 41, 42]. While *ccr4Δ* decreased TORC1 signaling, loss of individual PUF proteins had no effect (Fig 1D). Collectively, these data further support the conclusion that Ccr4-Not activates TORC1 independently of its role in mRNA deadenylation. We next tested if this TORC1 inhibition was due to reduced mRNA expression for any of the TORC1 components. The mRNA levels for all TORC1 subunits (*TOR1*, *TOR2*, *LST8*, *KOG1*, and *TCO89*), and the downstream ribosomal S6 kinase (*YPK3*), were unaffected or increased slightly (*KOG1* and *TCO89*) by *ccr4Δ* (Fig 1E). Therefore, Ccr4-Not loss does not impair TORC1 signaling by reducing the mRNA expression of TORC1 constituents.

Ccr4-Not facilitates co-translational assembly of some large macromolecular complexes [43–45], so we next tested if *ccr4Δ* inhibited TORC1 by reducing TORC1 complex stability. To do so, we evaluated the association of the essential (and unique) TORC1 subunit Kog1 with the Lst8 subunit, which is an essential component shared between TORC1 and the additional TOR kinase complex TORC2 [46]. Kog1 immunoprecipitation (IP), but not the control IP, weakly co-precipitated Lst8 from WT extracts, while the *ccr4Δ* enhanced this association (Fig 1F). Intriguingly, the increased Kog1-Lst8 interaction correlated with higher overall Lst8 levels in *ccr4Δ* (Fig 1F), which is not due to increased *LST8* mRNA expression (Fig 1E). These data suggest *ccr4Δ* may increase Lst8 expression post-transcriptionally. Although the mechanism explaining this increased Lst8-Kog1 interaction is not yet known, these data indicate that Ccr4-Not loss does not disrupt TORC1 to inhibit its signaling.

### Ccr4-Not activates TORC1 independently of CWI pathway repression

**Fig 2:**
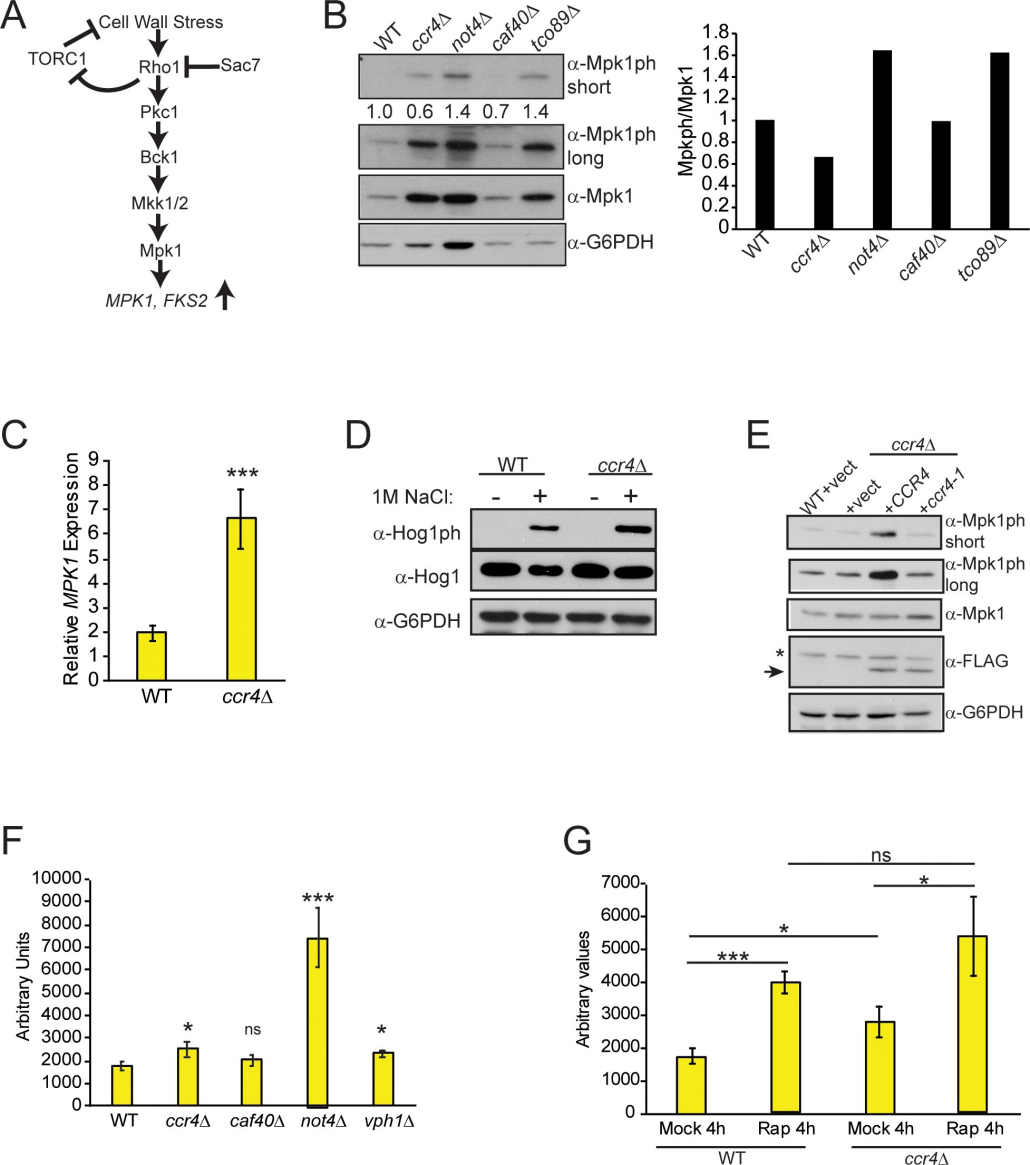
Ccr4-Not disruption deregulates expression of the CWI terminal kinase Mpk1. (A) Schematic of the CWI pathway adapted from [48]. (B) IB analysis of activated Mpk1 (Mpk1ph) and total Mpk1. Numbers reflect the Mpk1ph/Mpk1 ratio with WT set to 1 and the mutant Mpk1ph/Mpk1 ratio normalized to WT, and these data also are plotted in histogram format. Short and long film exposures for Mpk1ph are provided. Data are representative of three independent experiments. (C) RT-qPCR analysis of *MPK1* mRNA expression in WT and *ccr4Δ*. The data are the average and SD of three independent experiments with significance determined by Student’s t-test. ***-*p<0*.*005*. (D) IB analysis of activated p38/Hog1 (Hog1ph) in the indicated strains. As a control for Hog1 activation, WT and *ccr4Δ* were mock treated or treated with 1 M NaCl for 30 minutes to induce Hog1ph. Results are representative of three independent experiments. (E) WT and *ccr4Δ* expressing control vector, or vector expressing FLAG-tagged WT Ccr4 or the ccr4-1 mutant, were analyzed as indicated. Arrow denotes the Ccr4-specific band, and the asterisk denotes a cross-reactive protein. The data are representative of four independent experiments. (F) *In vitro* analysis of 20S proteasome catalytic activity from WT and the indicated mutants as described in the Methods. Data are the average and SD of three independent experiments. Significance was determined by comparing the level of 20S activity from each mutant to WT by pairwise Student’s t-test. * *p<0*.*05*; *** *p<0*.*005*; ns- not significant. (G) As in (F), except the WT and *ccr4Δ* cultures were mock treated or treated with 200 nM rapamycin for four hours. * *p<0*.*05*; *** *p<0*.*005*; ns- not significant.

The CWI pathway is activated by cell wall or membrane stress [47], and complete TORC1 inhibition is a known activator of CWI signaling [47]. CWI activation increases Rho1 GTPase activity, which signals through Pkc1 to ultimately activate the terminal MAPK Mpk1 to induce transcription of stress-responsive genes, including *MPK1* itself as well as genes such as *FKS2* (outlined in Fig 2A) [48]. Activated Rho1 also transiently inhibits TORC1 until cells adapt to the CWI activating stress [39], suggesting TORC1 and CWI exhibit mutually inhibitory cross-talk signaling. From WT and individual Ccr4-Not mutants grown in nutrient rich (YPD) media, we determined if Mpk1 signaling was affected by monitoring both activated (phosphorylated Mpk1, denoted Mpk1ph) and total Mpk1 [49]. For a comparison, we analyzed cells lacking the non-essential TORC1 subunit Tco89 whose loss decreases, but does not ablate, TORC1 signaling [50, 51]. The *tco89Δ* increased both Mpk1ph and total Mpk1 levels compared to WT, but after normalizing for the increase in Mpk1 expression the *tco89Δ* resulted only in a minor activation of Mpk1 (increased Mpk1ph/Mpk1 ratio) (Fig 2B). Both *ccr4Δ* and *not4Δ*, but not *caf40Δ*, similarly affected Mpk1ph and Mpk1 levels. While *not4Δ* increased Mpk1 activation similar to that detected for *tco89Δ*, the *ccr4Δ* resulted in a modest decrease in Mpk1 activation while *caf40Δ* had no effect (Fig 2B). These data demonstrate that while absolute levels of Mpk1ph and total Mpk1 are deregulated in Ccr4-Not and TORC1 mutants, overall Mpk1 activation remains only modestly increased (in *not4Δ* and *tco89Δ*), or it is decreased (in *ccr4Δ*) (Fig 2B). Ccr4-Not regulates post-transcriptional stability of *MPK1* mRNA via binding of the Ccr4 subunit to *MPK1* mRNA [22], so the increase in *ccr4Δ* of total Mpk1 likely reflects dysregulation of *MPK1* mRNA turnover. Indeed, we find that *ccr4Δ* increased *MPK1* mRNA expression (Fig 2C). However, this mechanism cannot fully account for the effect Ccr4-Not disruption has on Mpk1 expression since Ccr4 also binds *HOG1* mRNA (which encodes the p38 stress-inducible MAPK) [22, 52], yet *ccr4Δ* neither affected basal Hog1 protein levels nor its activation after osmostress (Fig 2D). Collectively, these data suggest that while reduced TORC1 signaling in *tco89Δ* and Ccr4-Not mutants deregulate Mpk1 expression, they have only minor effects on the overall action of Mpk1.

To further probe how *ccr4Δ* affects Mpk1 signaling, we analyzed Mpk1ph and total Mpk1 from WT and *ccr4Δ* vector control cells grown in nutrient defined SC selection media, along with *ccr4Δ* overexpressing either *CCR4* or the *ccr4-1* mutant. Surprisingly, in these growth conditions we did not detect an increase in Mpk1ph or total Mpk1 in the *ccr4Δ* vector control cells (Fig 2E). However, *ccr4Δ* overexpressing *CCR4* increased Mpk1ph without altering total Mpk1 levels, an effect dependent on its deadenylase activity since the *ccr4-1* mutant had no effect (Fig 2E). These results suggest that overexpressing the WT Ccr4 deadenylase may generate an mRNA degradation stress response that activates Mpk1 signaling. The data also suggest that the cellular growth environment has a significant role in determining if Ccr4-Not disruption affects Mpk1 expression since it does so under nutrient rich (YPD) conditions (Fig 2B), but not in nutrient defined SC media (Fig 2E).

TORC1 inhibition enhances Mpk1 signaling to induce expression of proteasomal subunits and chaperones, which enhances protein degradation and maintains cellular homeostasis [53]. Ccr4-Not also interacts with the proteasome [54], and Not4 is essential for correct proteasome assembly, catalytic activity, and cellular proteostasis [10, 11, 55]. Because Ccr4-Not loss represses TORC1 and deregulates Mpk1 expression, we analyzed the effect it has on proteasome activity by monitoring 20S core particle activity. As previously demonstrated, *not4Δ* dramatically increased 20S function (Fig 2F) [10, 11]. While *caf40Δ* had no effect, *ccr4Δ* caused a minor increase in 20S activity that resembled the increase caused by the vacuole V-ATPase mutant *vph1Δ*, which inhibits TORC1 (Fig 2F and Fig 1A). To test if *ccr4Δ* activates the proteasome by repressing TORC1, WT and *ccr4Δ* were mock-treated or treated with rapamycin to completely repress TORC1 activity. TORC1 inhibition increased proteasome activity in both WT and *ccr4Δ* to similar extents (Fig 2G). Therefore, the residual TORC1 signaling in *ccr4Δ* suffices to maintain TORC1-dependent proteasome repression even though Mpk1 expression is deregulated in these cells.

**Fig 3:**
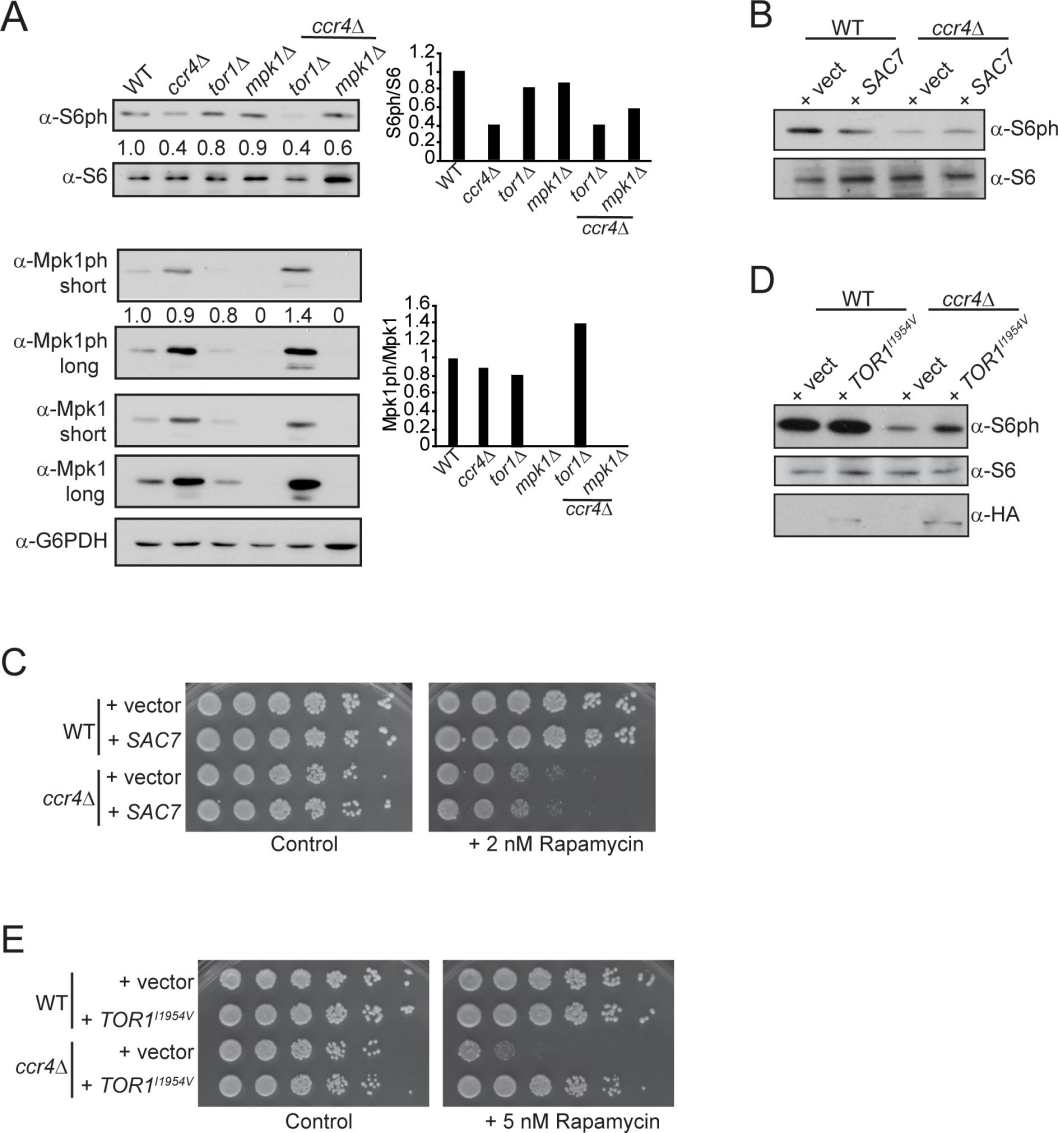
Ccr4-Not does not activate TORC1 signaling by repressing CWI signaling. (A) The WT and the indicated individual and combinatorial mutants were analyzed for both TORC1 signaling and Mpk1 activation. Numbers reflect either the S6ph/S6 or Mpk1ph/Mpk1 ratios with WT set to 1 and the individual mutants normalized to WT. These values also are plotted in histogram format. Short and long film exposures for Mpk1 are provided. The data are representative of three independent experiments. (B) WT and *ccr4Δ* transformed with control or high copy *SAC7* overexpressing vector were analyzed for S6ph and total S6 by IB. (C) Cells from (B) were cultured overnight to saturation before serially diluting equal numbers of cells five-fold and spotting to control media, or media containing 2 nM rapamycin. Plates were incubated for two days at 30°C. (D) As in (B) except cells were transformed with control vector or HA-tagged *TOR1*^*I1954V*^ expression vector. (E) Equal numbers of overnight cultures from (D) were five-fold serially diluted and spotted to control or 5 nM rapamycin plates and incubated at 30°C for two days.

TORC1 incorporates either the Tor1 or Tor2 kinase to mediate signaling, whereas the TORC2 complex utilizes only the Tor2 kinase. Consequently, a *tor1Δ* is viable and has minimal growth defects since Tor2 functions redundantly within TORC1 [46]. We next determined if combining *ccr4Δ* with *tor1Δ* or *mpk1Δ* synergistically impaired TORC1 signaling. While *ccr4Δ* inhibited TORC1 as expected, neither the *tor1Δ* nor *mpk1Δ* affected TORC1 activity. Furthermore, the *tor1Δ* also had no effect either on Mpk1 activation or expression (Fig 3A). Importantly, the *ccr4Δ tor1Δ* repressed both absolute S6ph levels and total S6 protein (Fig 3A). Although the S6ph/S6 ratio was similar between *ccr4Δ* and *ccr4Δ tor1Δ* (Fig 3A), the effects on total S6ph and S6 levels by *ccr4Δ tor1Δ* suggests the combinatorial mutant has a synthetic negative effect on TORC1 activity. This observation would be consistent with a previous report demonstrating the double mutant has a synthetic sick phenotype (which we address further below) [14]. Importantly, the *ccr4Δ tor1Δ* did not further deregulate Mpk1 expression or increase Mpk1 activation compared to *ccr4Δ* (Fig 3A). We note that the *ccr4Δ mpk1Δ* modestly increased total S6 levels, which resulted in a net reduction in S6ph (Fig 3A). The significance, if any, of this observation currently is unknown. Importantly, the *ccr4Δ mpk1Δ* does not restore TORC1 signaling compared to *ccr4Δ* (Fig 3A), thus demonstrating that deregulated Mpk1 expression does not mediate TORC1 inhibition in Ccr4-Not mutants.

The CWI pathway effector Rho1 GTPase transiently inhibits TORC1 when it is bound to GTP (the active signaling state) [39]. To exclude the possibility that *ccr4Δ* activates Rho1 independently of its role in the canonical CWI pathway to repress TORC1, WT and *ccr4Δ* cells were transformed with control vector, or a high copy vector overexpressing the *SAC7* genomic locus. Sac7 is a GTPase activating protein (GAP) that represses Rho1 signaling (Fig 2A) [48]. As expected, TORC1 activity in the *ccr4Δ* vector control was reduced relative to the WT control, while *SAC7* overexpression failed to restore TORC1 signaling in *ccr4Δ* (Fig 3B). Furthermore, *SAC7* overexpression also failed to restore *ccr4Δ* growth under TORC1 inhibitory conditions (Fig 3C). These data demonstrate that Ccr4-Not disruption does not enhance Rho1 signaling to repress TORC1. In contrast, a Tor1 gain of function mutant (Tor1^I1954V^) expressed in *ccr4Δ* partially restored TORC1 function (Fig 3D) [56]. This partial TORC1 rescue is due to competition of the Tor1^I1954V^ mutant with endogenous WT Tor1 and Tor2 kinases for incorporation into TORC1. However, the modest increase in TORC1 signaling suffices to restore growth in TORC1 inhibitory conditions (Fig 3E). Collectively, our results demonstrate that neither Mpk1 deregulation, nor Rho1 activation, explain the TORC1 inhibition in the Ccr4-Not mutant.

### Ccr4-Not regulates V-ATPase stability and function to activate TORC1

**Fig 4:**
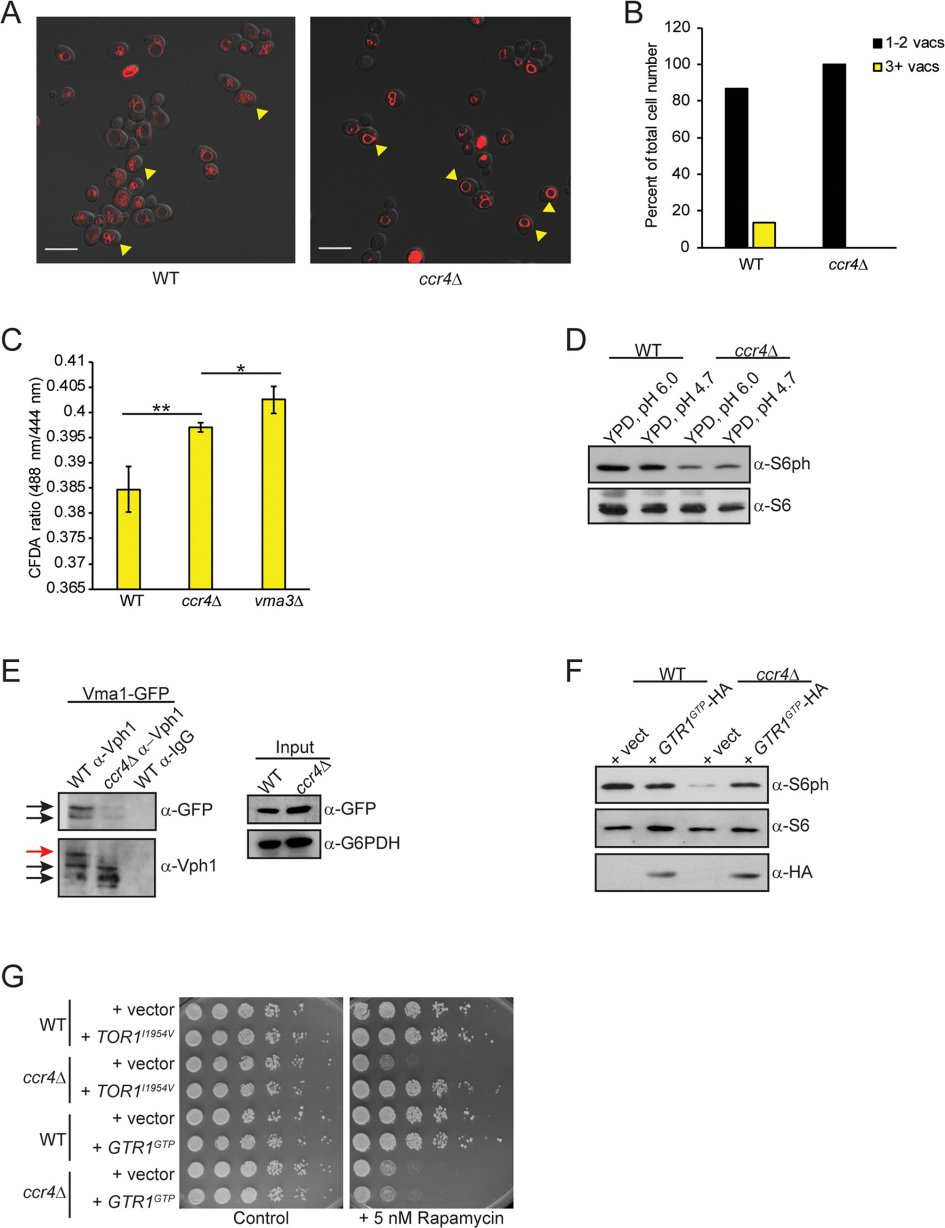
Ccr4-Not promotes the function of the vacuole V-ATPase to activate TORC1. (A) Mid-log phase WT and *ccr4Δ* cultured in YPD were stained with the vacuole-specific dye FM 4–64 and analyzed by confocal microscopy. Arrowheads in the WT panel indicate cells with three or more vacuoles, while the arrowheads in the *ccr4Δ* panel indicate cells with a single vacuole. Scale bar indicates 10 μm. (B) Quantification of vacuole number from (A) with a total of 186 individual WT and 281 *ccr4Δ* cells counted. Images from three independent staining experiments with randomly chosen fields of cells were quantified, and only cells with distinguishable vacuoles were enumerated. (C) WT, *ccr4Δ*, and *vma3Δ* were grown in YPD to mid-log phase, stained with 6-CFDA, and then equivalent cell numbers were analyzed on a Molecular Device plate reader with excitations performed at 444 nm and 485 nm, and emissions measured at 538 nm. The indicated 444/485 ratios are the average and SD of three independent experiments with the indicated pairwise Student’s t-test determining significance. *-*p< 0*.05; **-p< 0.*01*. (D) IB analysis of TORC1 signaling in WT and *ccr4Δ* cultured in the indicated media. (E) The α-Vph1 and α-IgG control IPs were performed from WT and *ccr4Δ* extracts expressing Vma1-GFP. IP samples then were split into two and analyzed by IB with the indicated antibodies. (F) TORC1 activity was determined by IB of extracts from WT and *ccr4Δ* transformed with control or Gtr1^GTP^ expression vectors. (G) Cells from (F) were cultured overnight to saturation, and then equal cell numbers were five-fold serially diluted and spotted to control or 5 nM rapamycin plates. Plates were incubated for two days at 30°C.

Ccr4-Not mutants previously were identified in a genetic screen for vacuole regulators [57]. Because the vacuole has a crucial role in TORC1 activation [23, 28], we tested if *ccr4Δ* altered vacuole function. WT and *ccr4Δ* grown in YPD were stained with the vacuole-specific dye FM 4–64, analyzed by confocal microscopy, and the vacuole number per cell was enumerated. WT cells had 1–2 vacuoles per cell (~85% of total cells), with a smaller number of cells (~15%) having three or more vacuoles (Fig 4A and Fig 4B). In contrast, *ccr4Δ* had no more than two vacuoles per cell, with most cells containing only a single large vacuole (Fig 4A and Fig 4B). Cells containing single large vacuoles indicate defects in vacuole acidification, which is a process that requires functional vacuole V-ATPase activity [58]. To test if *ccr4Δ* altered vacuole acidification, WT and *ccr4Δ* were stained with 6-carboxyfluorescein diacetate (6-CFDA) that is imported into the vacuole where it fluoresces as a function of decreasing pH. This fluorescence was quantified using a ratiometric assay that allows a relative assessment of vacuole acidity [59]. As a control, we analyzed a *vma3Δ* mutant, which ablates all cellular V-ATPase activity [60]. The *vma3Δ* resulted in a higher fluorescence ratio relative to WT, which indicates defective vacuole acidification, while the *ccr4Δ* had an intermediate effect (Fig 4C). These data demonstrate that *ccr4Δ* increases vacuole pH, which suggests that Ccr4-Not disruption impairs V-ATPase activity to inhibit TORC1.

A V-ATPase defect in Ccr4-Not mutants could inhibit TORC1 indirectly by increasing vacuole pH and reducing the level of free intra-vacuole amino acids required to activate TORC1 [31]. Alternatively, the V-ATPase interacts with Gtr1 in the EGO complex to activate TORC1 [28], so V-ATPase defects could impair TORC1 activation directly by reducing Gtr1-dependent activation. Because growth of V-ATPase mutants in acidic media partially suppresses V-ATPase phenotypes [61], we initially tested if an acidic growth environment could restore TORC1. WT and *ccr4Δ* were cultured in YPD media buffered to pH 4.7, or standard YPD, which is pH ~ 6.0. The acidic environment had no discernible effect on WT TORC1 signaling, while the acidic pH failed to restore TORC1 signaling in *ccr4Δ* (Fig 4D). Therefore, an acidic growth environment is insufficient to restore TORC1 activity in the Ccr4-Not mutant. Next, we determined if Ccr4-Not disruption altered V-ATPase stability by immunoprecipitating (IP) the V_0_ Vph1 subunit from WT and *ccr4Δ* cells expressing the V_1_ subunit Vma1 as a GFP fusion (Vma1-GFP). The α-Vph1 and α-IgG control IPs were split into two and either probed with α-GFP to detect Vma1 co-association, or probed with α-Vph1 to assess IP efficiency. The α-Vph1 IP from WT extracts co-precipitated two specific Vma1 bands, with the uppermost band likely being a post-translationally modified Vma1 as it is extensively modified [62–64]. The *ccr4Δ* dramatically reduced Vma1 co-association with Vph1, indicating that V-ATPase V_0_-V_1_ interactions are less stable in *ccr4Δ* (Fig 4E). The Vph1-specific antibody has poor sensitivity, and Vph1 is undetectable in the input samples, so the α-Vph1 IB confirmed the IP efficiency between the samples (Fig 4E). Intriguingly, we do find that α-Vph1 IP from WT extracts results in multiple Vph1-specific bands, while *ccr4Δ* selectively reduces the presence of the uppermost Vph1-specific band (denoted by the red arrow in Fig 4E). As with Vma1, Vph1 is extensively post-translationally modified, including by ubiquitination, which promotes Vph1 turnover and vacuole membrane remodeling under TORC1 inhibitory conditions [62, 64–66]. These data indicate that Ccr4-Not promotes the V_0_-V_1_ interactions necessary for V-ATPase activity that is key for vacuole acidification and TORC1 activation. They also suggest the possibility that Ccr4-Not may promote V-ATPase stability by affecting the post-translational modification state of core V-ATPase subunits.

If *ccr4Δ* impairs V-ATPase function to inhibit TORC1, then bypassing the V-ATPase in *ccr4Δ* to activate TORC1 should restore signaling through this pathway. To directly test this possibility, we used the constitutively active Gtr1^Q65L^ (Gtr1^GTP^) mutant that bypasses the requirement for the V-ATPase in TORC1 activation [27, 28]. WT and *ccr4Δ* were transformed with control or Gtr1^GTP^-HA expression vectors, and then TORC1 signaling was analyzed. Gtr1^GTP^ in WT cells had no obvious effect on TORC1 activity, but Gtr1^GTP^ expression restored TORC1 signaling in *ccr4Δ* (Fig 4F). However, unlike the Tor1^I1954V^ mutant that partially restored *ccr4Δ* TORC1 activity and fully rescued its growth in the presence of rapamycin (Fig 3C and 3D and Fig 4G), Gtr1^GTP^ expression failed to restore *ccr4Δ* growth under these conditions (Fig 4G). Gtr1 acts at the vacuole surface upstream of TORC1 to activate TORC1 in response to nutrient sufficiency, while the Tor1^I1954V^ directly incorporates into the TORC1 complex. Therefore, these data suggest that Ccr4-Not may activate TORC1 by promoting V-ATPase function, and that it also may function at a step downstream of the V-ATPase to regulate TORC1 directly. This possibility would be consistent with the observation that *ccr4Δ* may affect the TORC1 complex directly since it increases Lst8-Kog1 interactions (Fig 1E).

### Ccr4-Not disruption remodels the transcriptome to enhance mitochondrial activity

**Fig 5:**
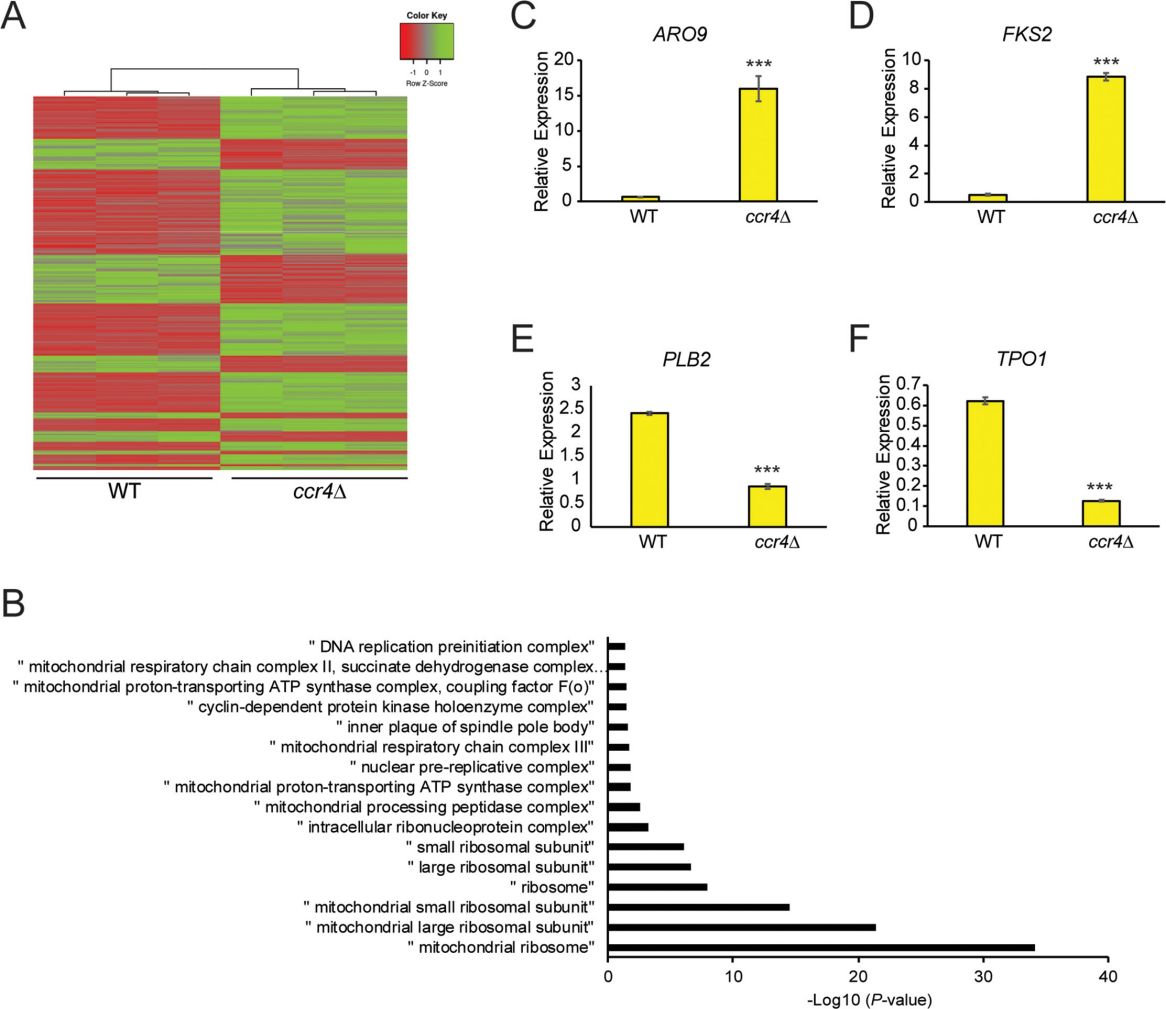
Ccr4-Not represses mitochondrial gene expression. (A) Heatmap of the differentially expressed genes between WT and *ccr4Δ* showing the individual replicates. (B) Analysis of the GO categories significantly upregulated in *ccr4Δ*. (C-F). RT-qPCR validation of the candidate upregulated (*ARO9* and *FKS2*) and downregulated (*PLB2* and *TPO1*) genes in *ccr4Δ* normalized to the expression of the housekeeping gene *SPT15*. Data are the average and SD of three independent experiments with significance determined by Student’s t-test. *** *p< 0*.*005*.

To further delineate how Ccr4-Not contributes to TORC1 regulation, we performed transcriptome sequencing (RNA-seq) from triplicate cultures of WT and *ccr4Δ* cells grown in nutrient rich YPD media. Since Ccr4 binds large numbers of mRNA transcripts to affect gene expression globally [22], we utilized a stringent 2-fold change in gene expression between WT and *ccr4Δ* to define those genes most affected by Ccr4-Not disruption. The expression of 499 total genes in *ccr4Δ* met these criteria, including 157 downregulated and 342 upregulated genes that are represented as a heatmap (Fig 5A, S1 Fig and S1 File). Gene ontology (GO) analysis of the downregulated genes revealed the single broad category “intracellular ribonucleoprotein complex” to be overrepresented (*p<0*.*027* by chi-square analysis) (S2 File). GO categories related to mitochondria, ribosomal synthesis, DNA replication, and components of the spindle pole body, were significantly overrepresented in the *ccr4Δ* upregulated gene set (Fig 5B and S3 File). These data confirm results from older microarray studies demonstrating that *ccr4Δ* increases expression of genes involved in metabolism, oxidative phosphorylation, and mitochondrial function [19, 20].

To confirm the integrity of the RNA-seq results, we selected from the dataset two highly induced (*ARO9* and *FKS2*) and repressed (*PLB2* and *TPO1*) genes, and their expression was verified by reverse transcription coupled with quantitative PCR (RT-qPCR). Ccr4 loss altered the expression of these genes as predicted by the transcriptome analysis (Fig 5C–5F), thus confirming the validity of our RNA-seq analysis. Although GO categories for the vacuole or V-ATPase were not overrepresented in *ccr4Δ*, two V-ATPase genes, *VMA6* and *VMA13*, were identified in the RNA-seq dataset as downregulated 2-fold by *ccr4Δ* (S1 File). To test if their reduced expression explained the TORC1 signaling defects, we cloned their genomic loci into high copy vectors and transformed both into WT and *ccr4Δ*. Simultaneous high copy expression of both V-ATPase subunits had no effect on TORC1 in WT or *ccr4Δ* (S2 Fig). Therefore, the *ccr4Δ* TORC1 signaling defects cannot be explained by reduced expression of these V-ATPase subunits.

**Fig 6:**
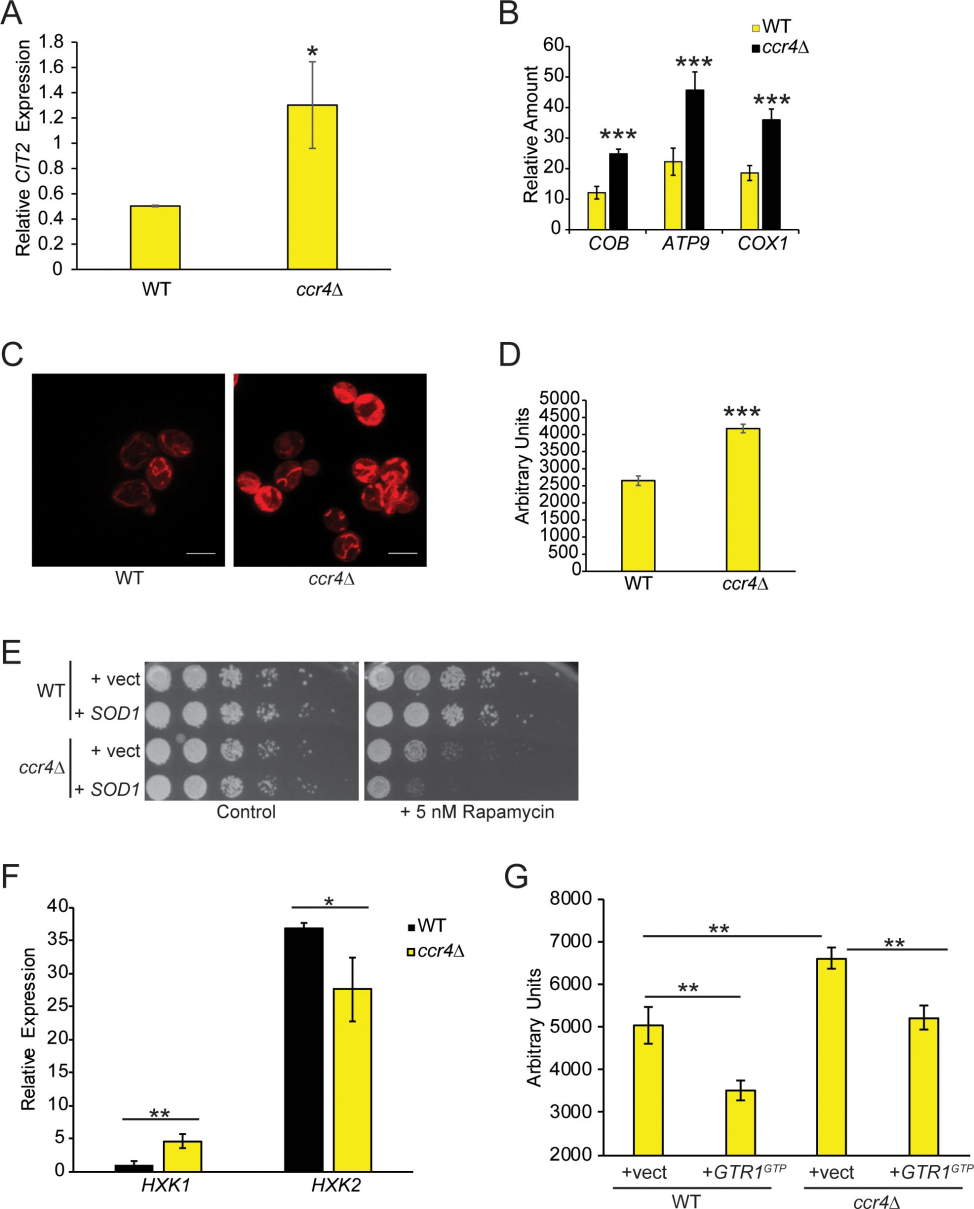
Ccr4-Not represses mitochondrial activity by activating TORC1 signaling. (A) RT-qPCR analysis of *CIT2* expression normalized to the housekeeping gene *SPT15*. Data are the average and SD of three independent experiments with significance determined by Student’s t-test. *- p< 0.*05*. (B) Relative quantification of mitochondrial DNA content. Genomic DNA from WT and *ccr4Δ* was used in qPCR with primers to the indicated mitochondrial genome-encoded genes normalized to the nuclear encoded gene *SPT15*. Data are the average and SD of three independent experiments with significance determined by Student’s t-test. ***- p<0.*005*. (C) WT and *ccr4Δ* were grown to mid-log phase in YPD, stained with Mitotracker, and analyzed by confocal microscopy. Scale bar indicates 5 μm. Data are representative of three independent experiments. (D) ROS levels were quantified by flow cytometry of WT and *ccr4Δ* DHE stained cells grown to mid-log phase in YPD. Data represent the average and SD three independent experiments with Student’s t-test determining significance. ***-p<0.*005*. (E) Equal numbers of overnight cultures of WT and *ccr4Δ* transformed with control or high copy vector overexpressing *SOD1* were five-fold serially diluted and spotted to control or 5 nM rapamycin plates. Plates were incubated at 30°C for two days. (F) RT-qPCR analysis of *HXK1* and *HXK2* expression in WT and *ccr4Δ*. Data are the average and SD of three independent experiments with significance determined by Student’s t-test. *- p<0.05; **- p<0.*01*. (G) ROS quantification of DHE stained mid-log phase WT and *ccr4Δ* cells transformed either with control or *GTR1*^*GTP*^ expression vector. Data represent the average and SD of three independent experiments, and significance was determined by Student’s t-test. **- p<0.*01*.

The enhanced mitochondrial gene expression, combined with the TORC1 and V-ATPase defects described above, suggests Ccr4-Not may induce a nutrient stress state that requires a compensatory increase in mitochondrial metabolism for cellular adaptation. TORC1 repression activates retrograde signaling to transcriptionally induce, via the transcription factors Rtg1 and Rtg3, nuclear-encoded mitochondrial genes including the gene *CIT2* [67, 68]. We analyzed *CIT2* levels by RT-qPCR and found that *ccr4Δ* significantly increased *CIT2* expression (Fig 6A), a result confirmed in the RNA-seq dataset (S1 File.). These data suggest *ccr4Δ* activates retrograde signaling to increase mitochondrial activity. To determine if this was indeed the case, we quantified the relative amount of mitochondrial DNA in WT and *ccr4Δ* by performing qPCR with primer sets to three distinct mitochondrial genome-encoded genes (*COB*, *ATP9*, and *COX1*) normalized to the signal for the nuclear encoded gene *SPT15*. The *ccr4Δ* increased the relative amounts of all three mitochondrial genes, which suggests *ccr4Δ* increases mitochondrial content (Fig 6B). We tested this directly by staining WT and *ccr4Δ* with Mitotracker and then analyzing cells by confocal microscopy. WT mitochondria exhibited their characteristic tubular morphology [69], while *ccr4Δ* mitochondria also were tubular but they stained more intensely and appeared thicker, which further supports the observation that they have increased mitochondrial content (Fig 6C). Dihydroethidium (DHE) staining to quantify reactive oxygen species (ROS) revealed that *ccr4Δ* increased ROS levels, which is consistent with their increased mitochondrial content (Fig 6D). Although excessive ROS causes toxicity, moderate ROS levels have signaling roles and promote hormetic responses that facilitate stress adaptation [70]. To determine if the ROS in *ccr4Δ* affects the cellular response to TORC1 stress, we transformed WT and *ccr4Δ* with control vector or vector overexpressing superoxide dismutase 1 (*SOD1*), which detoxifies oxygen radicals [71]. Cells were plated onto control plates, or plates containing a low (5 nM) rapamycin concentration. While *SOD1* had no effect on WT cells, *SOD1* overexpression in *ccr4Δ* further sensitized cells to TORC1 inhibition (Fig 6E). Therefore, the enhanced mitochondrial metabolism and ROS in *ccr4Δ* may contribute to a metabolic adaptive response required for coping with decreased TORC1 activity.

### Ccr4-Not loss upregulates mitochondrial metabolism to sustain TORC1 signaling

Mitochondrial metabolic remodeling involves changes to hexokinase expression such that the Hxk2 hexokinase expressed during glycolytic growth is downregulated while the Hxk1 isoform is upregulated [72]. Relative to WT, *ccr4Δ* decreased *HXK2* and increased *HXK1* mRNA expression (Fig 6F), thus further indicating that *ccr4Δ* enhances mitochondrial metabolism. To test if the increased mitochondrial metabolism is due to TORC1 repression, WT and *ccr4Δ* carrying control or GTR1^GTP^ expression vectors were stained with Mitotracker. WT cells expressing GTR1^GTP^ drastically reduced the mitochondrial signal compared to WT control vector cells but, surprisingly, we detected no obvious differences in Mitotracker staining between the WT control, *ccr4Δ* control, or *ccr4Δ* expressing Gtr1^GTP^ (S3 Fig). This result is in direct contrast to the same experiment performed in WT and *ccr4Δ* cells grown in YPD (Fig 6C). Although we currently cannot explain these different results, we believe they reflect differences due to the cellular nutrient environment since these experiments were performed in SC media (to select for plasmids), which is less nutrient rich than YPD. Regardless of these discrepancies, under these conditions Gtr1^GTP^ expression in both WT and *ccr4Δ* reduced mitochondrial ROS production relative to their respective controls. Importantly, GTR1^GTP^ expression reduced ROS in *ccr4Δ* to levels that resemble the WT vector control (Fig 6G). These data indicate that the increased ROS in *ccr4Δ* is a consequence of their reduced TORC1 signaling.

**Fig 7:**
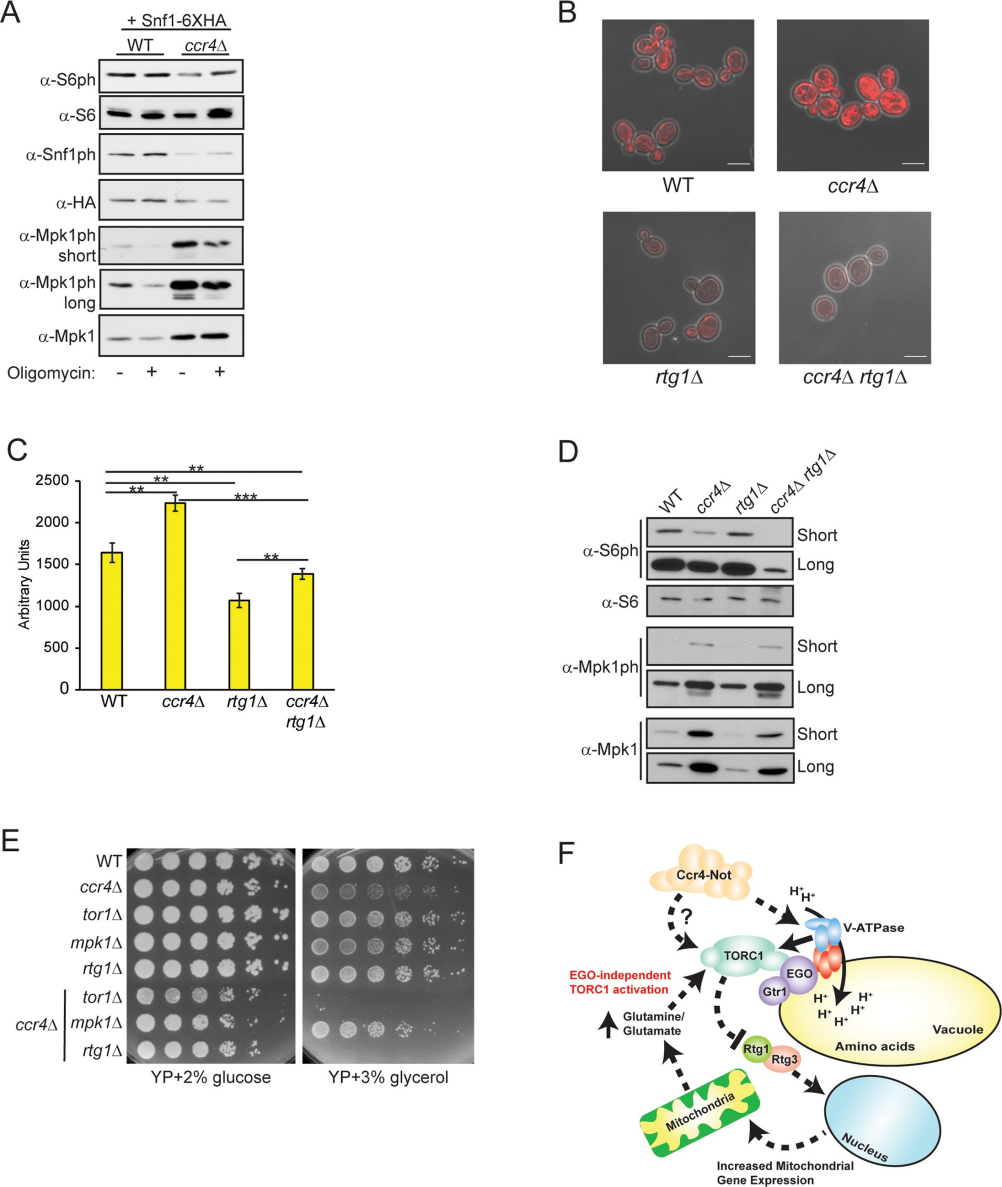
Mitochondrial upregulation in Ccr4-Not deficient cells sustains residual TORC1 signaling. (A) IB analysis for TORC1, Mpk1, and Snf1/AMPK activity in WT and *ccr4Δ* mid-log phase cells either mock treated or treated with 20 μM oligomycin for one hour. (B) Representative confocal microscopy analysis of WT, *ccr4Δ*, *rtg1Δ*, and *ccr4Δ rtg1Δ* cultured to mid-log phase in YPD and stained with Mitotracker. Scale bar indicates 5 μm. (C) ROS quantification of the indicated cells cultured to mid-log phase in YPD and stained with DHE. Data are the average and SD of three independent experiments with pairwise Student’s t-test determining significance. ** *p< 0*.*01*; *** *p< 0*.*005*. (D) Analysis of TORC1 and Mpk1 activation in WT, *ccr4Δ*, *rtg1Δ*, and *ccr4Δ rtg1Δ* grown to mid-log phase in YPD. Short and long film exposures are provided for clarity. (E) WT and the indicated mutants were cultured overnight, and then equal cell numbers were 5-fold serially diluted and spotted to the indicated plates. Plates were incubated for three days at 30°C. (F) Proposed model for how Ccr4-Not regulates TORC1 signaling. See text for details.

Metabolic stress activates the Snf1/AMPK kinase that represses TORC1, and it inhibits TORC1 in part by promoting formation of Kog1/Raptor bodies [73]. To test if *ccr4Δ* increased Snf1/AMPK activation, WT and *ccr4Δ* Snf1-6XHA expressing cells were mock treated or treated with the mitochondrial ATP synthase inhibitor oligomycin before assessing TORC1 activity. Mpk1 activation and Snf1/AMPK activation (phosphorylated Snf1/AMPK) also were analyzed. As demonstrated above, TORC1 signaling was impaired in the mock-treated *ccr4Δ*, while Mpk1 expression also was deregulated. Importantly, basal Snf1/AMPK phosphorylation was lower in *ccr4Δ*, which indicates decreased Snf1/AMPK activation (Fig 7A). This reduced Snf1/AMPK activation likely is a consequence of their enhanced mitochondrial metabolism and energy production. Although the oligomycin concentration used in these experiments failed to significantly activate Snf1/AMPK or repress TORC1 in either WT or *ccr4Δ*, it did reduce Mpk1ph in both suggesting mitochondrial respiration contributes to Mpk1 activation (Fig 7A). Snf1/AMPK also represses TORC1 through a separate mechanism involving the PAS kinase Psk1 that phosphorylates the stress granule factor Pbp1. Pbp1 then sequesters TORC1 into cytoplasmic stress granules, which inhibits TORC1 signaling [74]. Because a *pbp1Δ ccr4Δ* suppresses some *ccr4Δ* phenotypes [75], we tested if Ccr4-Not disruption inhibits TORC1 through Pbp1 by analyzing TORC1 in WT, *ccr4Δ*, *pbp1Δ*, and *ccr4Δ pbp1Δ*. The *ccr4Δ pbp1Δ* failed to restore TORC1 activity as would be predicted if *ccr4Δ* caused sequestration of TORC1 into stress granules (S4 Fig). Additionally, the double mutant had no effect on Mpk1ph, demonstrating that the deregulated Mpk1 expression in *ccr4Δ* is independent of the stress granule pathway (S4 Fig). Therefore, TORC1 inhibition in Ccr4-Not mutants cannot be explained by activation of either Snf1/AMPK dependent inhibitory pathways.

Mitochondria can serve as a metabolic source of amino acids since the TCA cycle intermediate α-ketoglutarate is used to synthesize glutamate via NADPH-dependent glutamate dehydrogenase. This mitochondrial-derived glutamate can be combined with ammonia via glutamine synthetase to also generate glutamine that is used as a nitrogen source for amino acid synthesis [76, 77]. A poorly understood glutamine-dependent, but EGO-independent, mechanism activates TORC1 in yeast, while glutamine also activates mammalian mTORC1 independently of the orthologous Ragulator complex [33, 78]. We next tested if the enhanced mitochondrial metabolism in *ccr4Δ* compensates for defective V-ATPase activity to activate TORC1. The *ccr4Δ* was combined with a *rtg1Δ* to prevent retrograde activation, and then WT, *ccr4Δ*, *rtg1Δ*, and *ccr4Δ rtg1Δ* grown in YPD were stained with Mitotracker. As seen previously, *ccr4Δ* increased mitochondrial content compared to WT, while *rtg1Δ* dramatically reduced overall mitochondrial number. Importantly, the *ccr4Δ rtg1Δ* completely prevented the mitochondrial increase mediated by *ccr4Δ* (Fig 7B). ROS production also reflected these mitochondrial changes since *rtg1Δ* reduced ROS levels to below that of WT, while the *ccr4Δ rtg1Δ* completely suppressed the increased ROS due to *ccr4Δ* (Fig 7C). We next assessed whether retrograde activation contributed to either TORC1 signaling or Mpk1 regulation in these cells. As described above, the *ccr4Δ* reduced TORC1 activity and deregulated Mpk1 expression, while the *rtg1Δ* had no effect on TORC1 but it did reduce total Mpk1 expression (Fig 7D). Importantly, the *ccr4Δ rtg1Δ* decreased TORC1 signaling to a level substantially below that of *ccr4Δ* without affecting Mpk1 expression (Fig 7D). These results clearly indicate that preventing mitochondrial upregulation in Ccr4-Not deficient cells suppresses TORC1 signaling further. To probe this metabolic connection *in vivo*, we assessed growth of WT, *ccr4Δ*, *tor1Δ*, *mpk1Δ*, *rtg1Δ*, and the *ccr4Δ* combined with each of these mutants on both control (YP + 2% glucose, YPD media) or YP + 3% glycerol media. Glycerol is a non-fermentable carbon source that requires mitochondrial metabolism to support cell growth [79]. Relative to WT and the individual gene deletions, each of the double mutants grows less robustly on control media suggesting they have modest synthetic sick phenotypes (Fig 7E). Importantly, on glycerol media the *ccr4Δ* caused a mild growth phenotype relative to WT (indicated by reduced colony size), while growth of the *tor1Δ*, *mpk1Δ*, and *rtg1Δ* was similar to WT. Importantly, both the *ccr4Δ tor1Δ* and *ccr4Δ rtg1Δ*, but not the *ccr4Δ mpk1Δ*, were unable to grow on glycerol media (Fig 7E). These data indicate that Ccr4-Not, TORC1, and the Rtg1-activated retrograde pathway have partially overlapping roles in the *in vivo* regulation of mitochondrial metabolism. Although Mpk1 expression is upregulated in the absence of functional Ccr4-Not, the *mpk1Δ* had no effect on this mitochondrial metabolic response (Fig 7E). Collectively, we propose a model where decreased Ccr4-Not activity reduces the stability and function of the V-ATPase to impair vacuole-mediated amino acid dependent TORC1 activation. Consequently, this reduction in Ccr4-Not activity causes an adaptive increase in mitochondrial metabolism that provides a vacuole-independent source of amino acids required to sustain a minimal level of TORC1 signaling for cell growth and proliferation (Fig 7F).

## Discussion

Previous work has indicated that Ccr4-Not functions within the TORC1 pathway and that it also represses mitochondrial metabolism [14, 16, 18–20]. Yet whether these processes are linked, and how Ccr4-Not contributes to their regulation, has remained elusive. In this report we provide evidence that Ccr4-Not integrates these activities by promoting vacuole V-ATPase dependent TORC1 activation. In the absence of functional Ccr4-Not, V-ATPase V_0_-V_1_ interactions are less stable, which causes defective vacuole acidification and decreased TORC1 signaling. How Ccr4-Not promotes V-ATPase activity remains unclear, although our observations suggest one possibility could be that Ccr4-Not affects post-translational modification of V-ATPase subunits like Vph1. V-ATPase subunits are extensively modified, including many subunits that are ubiquitinated [62–64]. Although the contribution of ubiquitination to V-ATPase activity and/or stability remains mostly uncharacterized, a recent study determined that TORC1 inhibition increases Vph1 ubiquitination and turnover to promote vacuole membrane remodeling during nutrient stress adaptation [65]. While our data reveal that total Vph1 levels remain similar between WT and *ccr4Δ* (Fig 4E), there is a clear decrease in the highest mobility Vph1-specific band in *ccr4Δ*, suggesting Ccr4 loss may affect Vph1 modification. Because *ccr4Δ* and *not4Δ* both reduce TORC1 activity, TORC1 regulation involves the Ccr4-Not complex and not just the Ccr4 subunit alone. The *not4Δ* ubiquitin ligase mutant causes a well-established disruption in global ubiquitin homeostasis due to defective proteasome assembly and regulation [10, 11]. While the *ccr4Δ* effect on the proteasome is not as extensive as *not4Δ*, our data do indicate the *ccr4Δ* causes a minor increase in proteasome activity, which could indicate modest proteasome dysfunction. Therefore, one explanation for our observations could be that Ccr4 has a minor role in Not4 regulation of the proteasome. Loss of Ccr4 then might affect proteasome-dependent ubiquitin recycling that reduces V-ATPase subunit ubiquitination, especially since ubiquitin levels are tightly regulated [80]. If V-ATPase ubiquitination promotes its stability and/or activity, Ccr4-Not mutants that impair ubiquitin homeostasis could decrease vacuole acidification and TORC1 activation by altering V-ATPase subunit ubiquitination.

Additionally, *not4Δ* exhibits synthetic sick and/or lethal phenotypes with mutations in several ubiquitin ligases that regulate the endocytic compartment [81]. These genetic data suggest Not4 may ubiquitinate endocytic-specific substrates, a definite possibility since few Not4 substrates have been identified. V-ATPase activity is critical for function of the endocytic pathway [82]. If Not4 influences V-ATPase subunit ubiquitination, then Ccr4-Not could have broader roles in the regulation of the endocytic compartment. Since Ccr4 is positioned near Not4 in the Ccr4-Not complex [83], Ccr4 might participate in substrate selection by Not4 that has a role in endocytic regulation. Such a possibility would explain why *ccr4Δ* impairs V-ATPase activity and inhibits TORC1. Alternatively, if the V-ATPase is co-translationally assembled, then Ccr4-Not disruption may destabilize the V-ATPase by inhibiting this process since Ccr4-Not controls co-translational assembly of other large macromolecular complexes [43, 44].

Regardless of how Ccr4-Not promotes V-ATPase activity, V-ATPase disruption is a known TORC1 inhibitor [28]. Expressing Gtr1^GTP^, which bypasses the V-ATPase to activate TORC1, fully restored TORC1 signaling in *ccr4Δ*. This result further supports our model that Ccr4-Not activates TORC1 through V-ATPase regulation. Ccr4-Not regulation of the V-ATPase also explains why some Ccr4-Not mutants have vacuole morphology defects since V-ATPase mediated vacuole acidification regulates vacuole morphology [57]. Our data do not support the alternative possibilities that Ccr4-Not loss activates known TORC1 inhibitory pathways, including those mediated by Mpk1, Rho1, or Snf1/AMPK signaling. Because the V-ATPase also activates PKA signaling through the Arf1 GTPase [28], Ccr4-Not may promote V-ATPase activity to coordinate both TORC1 and PKA signaling, a possibility that will need to be explored in future studies. If this occurs, then Ccr4-Not could be a key regulatory node for integrating nitrogen and glucose-dependent metabolic regulation.

Although Gtr1^GTP^ expression restored TORC1 signaling in *ccr4Δ*, it failed to rescue *ccr4Δ* growth under TORC1 inhibitory conditions. These data suggest that Ccr4-Not also acts downstream of the V-ATPase to regulate TORC1. This control may be at the level of TORC1 structure and/or TORC1 subunit availability, a possibility for which we have circumstantial evidence since *ccr4Δ* increases both Lst8 expression and its interaction with TORC1. Structural studies of the human mTORC1 complex reveal that mLst8 binds to the ATP-binding cleft to limit ATP binding and prevent mTORC1 from phosphorylating non-specific substrates [84]. The increased Lst8 association with Kog1 we detect in *ccr4Δ* might further restrict ATP binding, which could reduce TORC1 kinase activity. How Ccr4-Not disruption increases Lst8 protein levels currently is not understood, but it likely occurs post-transcriptionally since *ccr4Δ* does not increase *LST8* mRNA expression. Intriguingly, both Lst8 and Kog1 are WD40-repeat containing proteins, and the WD40 repeats in Kog1 bind ubiquitin under stress conditions to stabilize the complex [85]. Whether yeast Lst8 binds ubiquitin is unknown, but K63-linked polyubiquitination of mammalian mLst8 does promote preferential incorporation of mLst8 into mTORC1 over mTORC2 [86]. Therefore, some precedent for ubiquitin signaling is known to drive both yeast TORC1 and mammalian mTORC1 signaling. If, as discussed above, Lst8 binding to ubiquitin affects its stability or TORC1 incorporation, then Ccr4 loss could alter Not4-dependent ubiquitin homeostasis to more directly control TORC1 signaling. More detailed future studies will be required to refine our understanding of how Ccr4-Not regulates TORC1 activity.

TORC1 inhibition in Ccr4-Not mutants is predicted to generate a nutrient stress response that activates retrograde signaling to increase mitochondrial metabolism [67, 68], a result we demonstrate does occur. Retrograde activation in Ccr4-Not mutants likely functions as an adaptive mechanism to compensate for reduced V-ATPase dependent TORC1 activation since blocking retrograde signaling further reduces TORC1 activity. We believe Ccr4-Not mutants enhance mitochondrial metabolism to sustain a minimal level of TORC1 signaling required for growth and proliferation. Such a mechanism most likely involves diverting some of the mitochondrial α-ketoglutarate into glutamate and glutamine biosynthetic pathways. These vacuole-independent amino acid pools then could activate TORC1 independently of the V-ATPase, or perhaps enhance the ability of the impaired V-ATPase to activate TORC1. Evidence exists for vacuole EGO-independent TORC1 activation mechanisms in yeast [33], and an analogous process also occurs in mammals [78]. This mechanism also would be consistent with the increased sensitivity *ccr4Δ* has to TORC1 inhibition when the *SOD1* superoxide dismutase is overexpressed. Sod1 enzymatically stabilizes the casein kinase I-gamma (CK1γ) isoforms Yck1 and Yck2 (Yck1/2) to promote glucose and amino acid metabolism, and CK1γ also represses mitochondrial respiration [87]. Increasing Sod1 levels in *ccr4Δ* likely enhances CK1γ signaling to repress the mitochondrial upregulation required for *ccr4Δ* to bypass V-ATPase dependent TORC1 activation. The increased ROS in *ccr4Δ* also may allow these cells to adapt to TORC1 stress through other means as well, since ROS increases stress resistance mechanisms [70].

Although Ccr4-Not clearly represses Mpk1ph and total Mpk1 levels, the relative level of Mpk1 activation remains only modestly affected in Ccr4-Not mutants. Most importantly, we confirmed this Mpk1 deregulation does not repress TORC1 since the combined loss of both Ccr4 and Mpk1 failed to restore TORC1 signaling. Ccr4-Not disruption likely deregulates Mpk1 in part by preventing post-transcriptional turnover of *MPK1* mRNA since its steady-state levels increase in *ccr4Δ*, and Ccr4 binds *MPK1* transcripts to promote their degradation [22]. However, Ccr4-Not likely regulates Mpk1 through additional mechanisms that may include nutrient signaling since Ccr4-Not disruption deregulates Mpk1 expression in nutrient rich, but not nutrient defined, media. The mechanisms underlying this difference, and their impact on Mpk1 regulated stress pathways will need to be delineated in future studies. Collectively, we provide evidence that Ccr4-Not stimulates TORC1 signaling through the vacuole V-ATPase that activates TORC1 in response to amino acid sufficiency. Upon Ccr4-Not inactivation, cells upregulate mitochondrial metabolism in part to compensate for this defect in TORC1 activity that then sustains a minimum level of TORC1 signaling to promote cell growth and proliferation. The V-ATPase activates mTORC1 in mammalian cells [31], and Ccr4-Not has poorly understood roles in mammalian cellular metabolism [88–90]. Our studies suggest the possibility that these mechanisms could be conserved in human cells to allow Ccr4-Not to activate mTORC1. Such a possibility could explain why Ccr4-Not has such critical roles in cell growth and proliferation, and why its disruption plays an important role in a wide variety of diseases and developmental disorders.

## Materials and methods

### Yeast strains and culture conditions

Yeast strains are derivatives of the BY4741 background and are listed in S1 Table. All yeast growth media components were purchased from US Biologicals and Research Products International. Cells were grown to mid-log phase (OD_600_ = 0.8–1.2) for all experiments. Experiments performed in nutrient rich media utilized YPD (1% yeast extract, 2% peptone, 2% dextrose), while those experiments where selection for plasmid maintenance was required were performed in synthetic complete (SC) media (0.2% yeast nitrogen base, 0.5% ammonium sulfate, 2% dextrose, 0.19% amino acid dropout mix). To make the appropriate SC selective media, all nutrients lacking in the dropout mix were added back except the nutrient that allowed for plasmid selection. Yeast genetic manipulations to generate integrated epitope tags or gene deletions were performed following standard procedures as previously described [91].

### Chemical reagents and proteasome assay

Rapamycin (catalog 12–921) was purchased from Tocris/Fisher Scientific, while the 5(6)-carboxyfluorescein diacetate (CFDA) (catalog 21879), dihydroethidium (DHE) (catalog D7008), and oligomycin (495455) were purchased from Sigma-Aldrich. The Proteasome 20S Activity Assay Kit (catalog MAK172) was purchased from Sigma-Aldrich. Phostag reagent was purchased from Wako Chemical.

### Cloning

All plasmids and PCR primers used in this study are listed in S2 Table and S3 Table. Plasmids overexpressing *SAC7*, *VMA6*, and *VMA13* were generated by amplifying 300 base pairs upstream of their translational start site and 100 base pairs downstream of the translational stop using Q5 high fidelity DNA polymerase (New England Biolabs). PCR fragments were then cloned into high copy vectors pRS425 or pRS426. The Gtr1GTP expression vector was generated by amplifying Gtr1^Q65L^ as a C-terminal mono-HA tag fusion from plasmid pMB1394 [27], and then the PCR fragment was cloned into plasmid p416ADH [92]. The Ccr4 and ccr4-1 C-terminal mono-FLAG expression plasmids were generated using Q5 enzyme with plasmid templates pADHCCR4 and pADHccr4-1, and the resulting PCR fragments then cloned into p415ADH [92]. All plasmids were sequenced to confirm cloned products were correct.

### Whole cell extract preparation and immunoprecipitations

Yeast total cell extracts were prepared as described previously [93]. Briefly, cells were harvested by centrifugation, washed once with ddH_2_0, and then the pellets frozen at -80°C. For standard SDS-PAGE analysis, cell pellets were lysed by bead beating in lysis buffer (300 mM NaCl, 10% glycerol, 10 mM Tris pH 8.0, and 0.1% NP-40) containing protease and phosphates inhibitors and 1 mM DTT. All standard immunoblot analyses were performed using 30 μg total cell extract. For the immunoprecipitation experiments, extracts were prepared in the same buffer but containing 150 mM NaCl instead. Lysed extracts were clarified by centrifugation at 4°C for 15 minutes, and then the supernatants were quantified by Bradford assay. Immunoprecipitations were performed using 750 μg-1 mg total cell extracts for four hours to overnight incubation at 4°C with rotation. Immune complexes were isolated with Protein A-conjugated agarose beads, washed extensively with extraction buffer, and then resuspended in 2X SDS-loading buffer and resolved by SDS-PAGE. For input samples, 30 μg cell extracts were analyzed. Immunoblots were quantified using ImageJ. All experiments were independently repeated a minimum of three or more times.

### Antibodies

The antibodies used are as follows: rabbit α-RPS6 (ab40820) and rabbit α-Vph1 (ab113683) from Abcam; rabbit α-Phospho S6 (#2211), rabbit α-Phospho-p44/42 MAPK (#4370), and rabbit α-Phospho-AMPKa (#9211) from Cell Signaling Technology; mouse α-Mpk1 (sc-133189), mouse α-Hog1 (sc-165978), mouse α-Myc clone 9E10 (sc-40), and mouse α-HA clone F-7 (sc-7392) from Santa Cruz Biotechnology; mouse α-GFP (Y1030) from UBPBio; and rabbit α-G6PDH (A9521) from Sigma-Aldrich.

### Cell staining and confocal microscopy

For analysis of ROS, cells were cultured overnight to OD_600_ = 0.8 and 1 mL of culture was harvested, centrifuged to remove the media, and then resuspended in 200 μL of PBS containing 20 μM DHE for 15 minutes at 30°C. Samples then were analyzed on a BD Accuri C6 flow cytometer. Vacuole staining was performed by culturing cells overnight to OD_600_ = 0.8, and then removing 1 mL of culture and adding FM 4–64 to 1 mg/mL for 15–30 minutes before analyzing by confocal microscopy. To detect mitochondria, cells were cultured and harvested as described immediately above, but then they were resuspended in 10mM HEPES buffer (pH 7.4) with 5% glucose. Mitotracker Red FM was added to a final concentration of 100 nM and cells were incubated in a shaker for 15–30 minutes at 30°C. Stained cells then were visualized on a Zeiss confocal microscope using a 63X oil objective with Z-stacks (4–5) taken for each image. To analyze vacuole pH, overnight cell cultures at OD_600_ = 0.8 were harvested, media was removed, and cells washed in ddH_2_0. Cells then were resuspended in YPD containing 50 mM citric acid adjusted to pH 3.5 and 6-CFDA was added to a final concentration of 20 μm for 30 minutes in a 30°C shaker. Cells were cooled on ice before centrifuging and removing the media. The cell pellet was washed twice using pre-cooled YPD, and then each sample was resuspended to the same OD_600_ in a 96 well plate. The plate was analyzed on a Molecular Device plate reader using excitations at 444 nm and 485 nm with emissions captured at 538 nm.

### RNA sequencing and bioinformatics analysis

Total RNA was isolated by hot phenol extraction from mid-log phase WT and *ccr4Δ* cultures grown in YPD in triplicate, and RNAs then were treated with DNase I to get rid of contaminating genomic DNA. The purified RNA samples were submitted to the University of Tennessee Molecular Resource Center where they were analyzed on an Agilent Bioanalyzer to assess the RNA quality with RIN numbers meeting a minimum ≥8.0. After passing this initial screening, 250 ng of total RNA was used to prepare libraries for sequencing using the Qiagen Qiaseq FastSelect rRNA depletion kit to eliminate rRNA. The libraries were then prepared using the NEB Ultra II directional RNA-seq kit for Illumina and then amplified for 15 cycles as the final step of library preparation. Libraries were quantified using data from the Agilent Bioanalyzer and a Qbit fluorometer, and then they were pooled in equimolar amounts. Following pooling, the library pools were sized to a target range of 300 bp-700 bp using AMPpure beads. The sized libraries were examined on an Agilent High Sensitivity DNA chip, quantified using a Q-bit fluorometer, and used for sequencing on the Illumina NextSeq platform. All fastq files then were gathered from the sequencer, and quality assurance was performed using FASTQC. Reads were trimmed to remove any nucleotide with a PHRED score < Q20, and the trimmed FASTQ files were aligned to the yeast coding fasta reference library using RNA STAR. Once aligned, the SAM files were collected and mined for the read count information of each gene present in the reference file. Read counts were normalized using the Counts per Million (CPM) method across the entire experiment. Principle component analysis and Pearson’s coefficient plots were performed on the normalized transcriptome profile. A Wilcoxon’s t test was used to determine significance between conditions, and all genes that failed to yield a p-value greater than 0.05 were removed. Benjamini and Hochberg false discovery rate was performed on the trimmed gene list, and all genes failing to yield a false discovery rate of less than 0.05 were discarded. The final significant differential gene list was loaded into R to generate heatmaps. The gene targets were loaded into GO term finder (version 0.86) for biological function and process categorical analysis. The summary statistics for all graphical data are presented in S4 File.

## Supporting information

S1 Fig Pearson’s correlation analysis of the WT and *ccr4Δ* RNA-seq data. CPM normalized read counts were log2 transformed, and the Pearson’s correlations and scatterplots of transformed data were generated using corrgram package in R.
(TIF)

S2 Fig *VMA6* and *VMA13* overexpression does not restore TORC1 signaling in *ccr4Δ*. WT and *ccr4Δ* were transformed with high copy control vector or vectors expressing both *VMA6* and *VMA13*. TORC1 signaling was analyzed from mid-log phase cultures. Data are representative of three independent experiments.
(TIF)

S3 Fig Mitochondrial analysis in WT and *ccr4Δ*. WT and *ccr4Δ* were transformed with control vector or vector expressing Gtr1^GTP^. Cells were grown to mid-log phase and then stained with Mitotracker, and then analyzed by confocal microscopy. Scale bar indicates 5 μm.
(TIF)

S4 Fig Pbp1 loss does not rescue TORC1 signaling in Ccr4-Not deficient cells. WT, *ccr4Δ*, *pbp1Δ*, and *ccr4Δ pbp1Δ* were analyzed for both TORC1 and Mpk1 signaling as indicated. Data are representative of three independent experiments.
(TIF)

S1 Table Yeast strains used in this study. (DOCX)

S2 Table Yeast plasmids used in this study. (DOCX)

S3 Table PCR primers used in this study. (DOCX)

S1 File RNA-seq results of all *ccr4Δ* differentially regulated genes. (XLSX)

S2 File Gene ontology (GO) analysis of all *ccr4Δ* downregulated genes. (XLSX)

S3 File Gene ontology (GO) analysis of all *ccr4Δ* upregulated genes. (XLSX)

S4 File Summary statistics for all graphical data. (XLSX)
