# Supplementary figures and images for: The Ccr4-Not complex regulates TORC1 signaling and mitochondrial metabolism by promoting vacuole V-ATPase activity
Source: PLoS Genet. 2020 Oct 16;16(10):e1009046. doi: 10.1371/journal.pgen.1009046 (PMC7592917; doi:10.1371/journal.pgen.1009046)

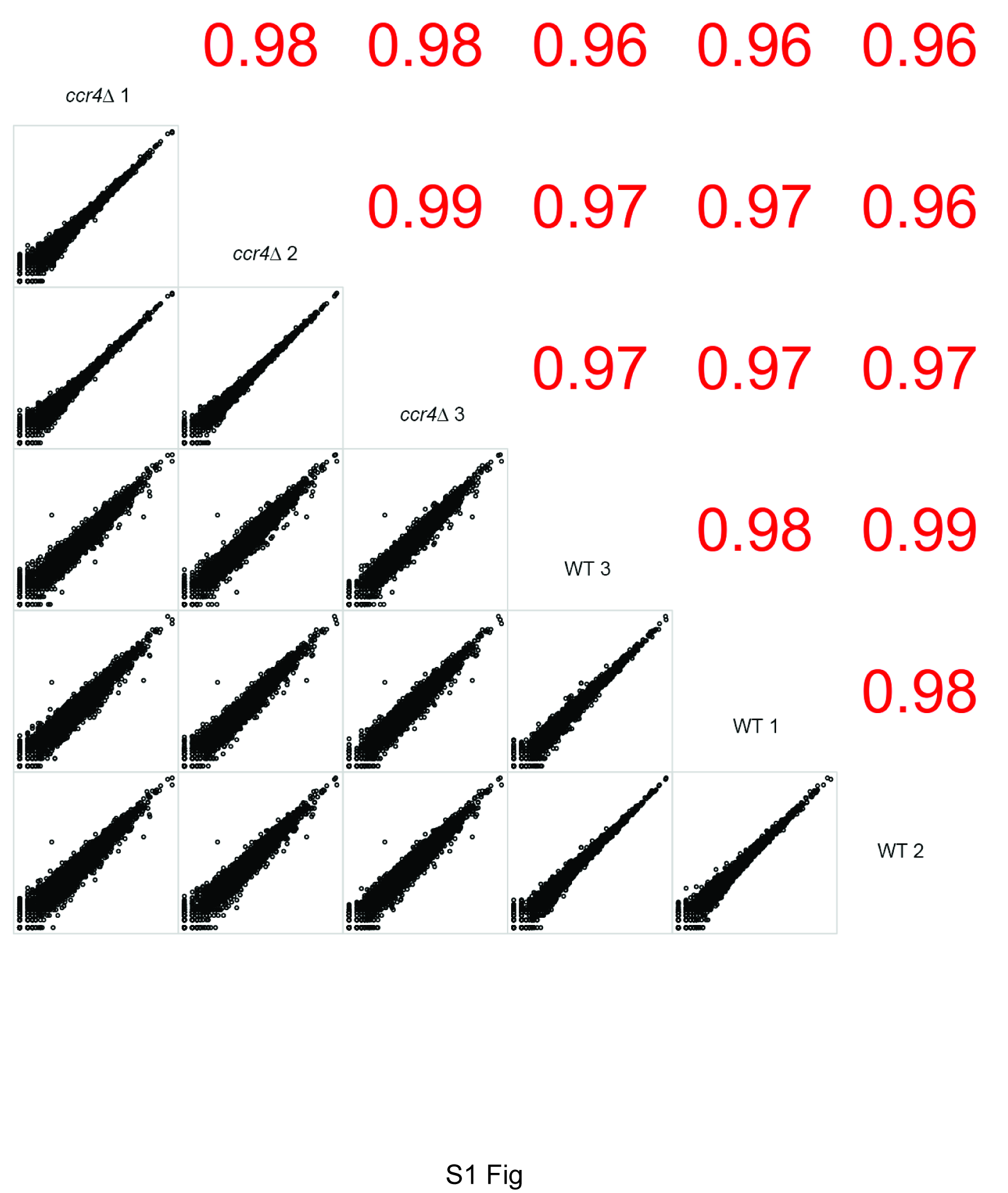

Supplement: S1 Fig — CPM normalized read counts were log2 transformed, and the Pearson’s correlations and scatterplots of transformed data were generated using corrgram package in R. (TIF) [file pgen.1009046.s001.tif]

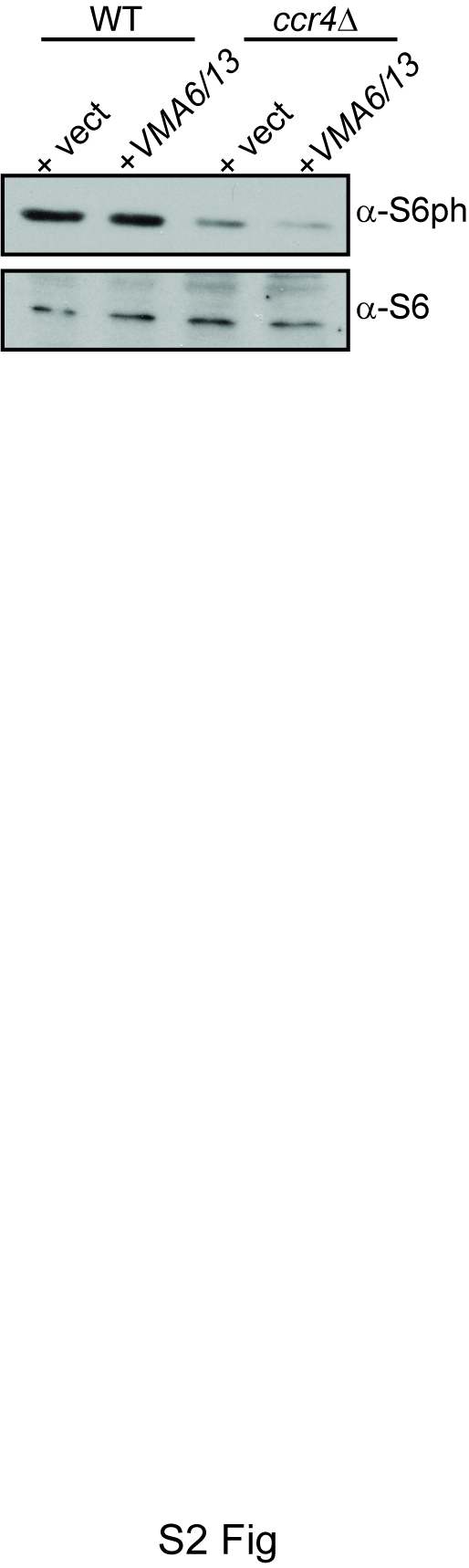

Supplement: S2 Fig — WT and ccr4Δ were transformed with high copy control vector or vectors expressing both VMA6 and VMA13. TORC1 signaling was analyzed from mid-log phase cultures. Data are representative of three independent experiments. (TIF) [file pgen.1009046.s002.tif]

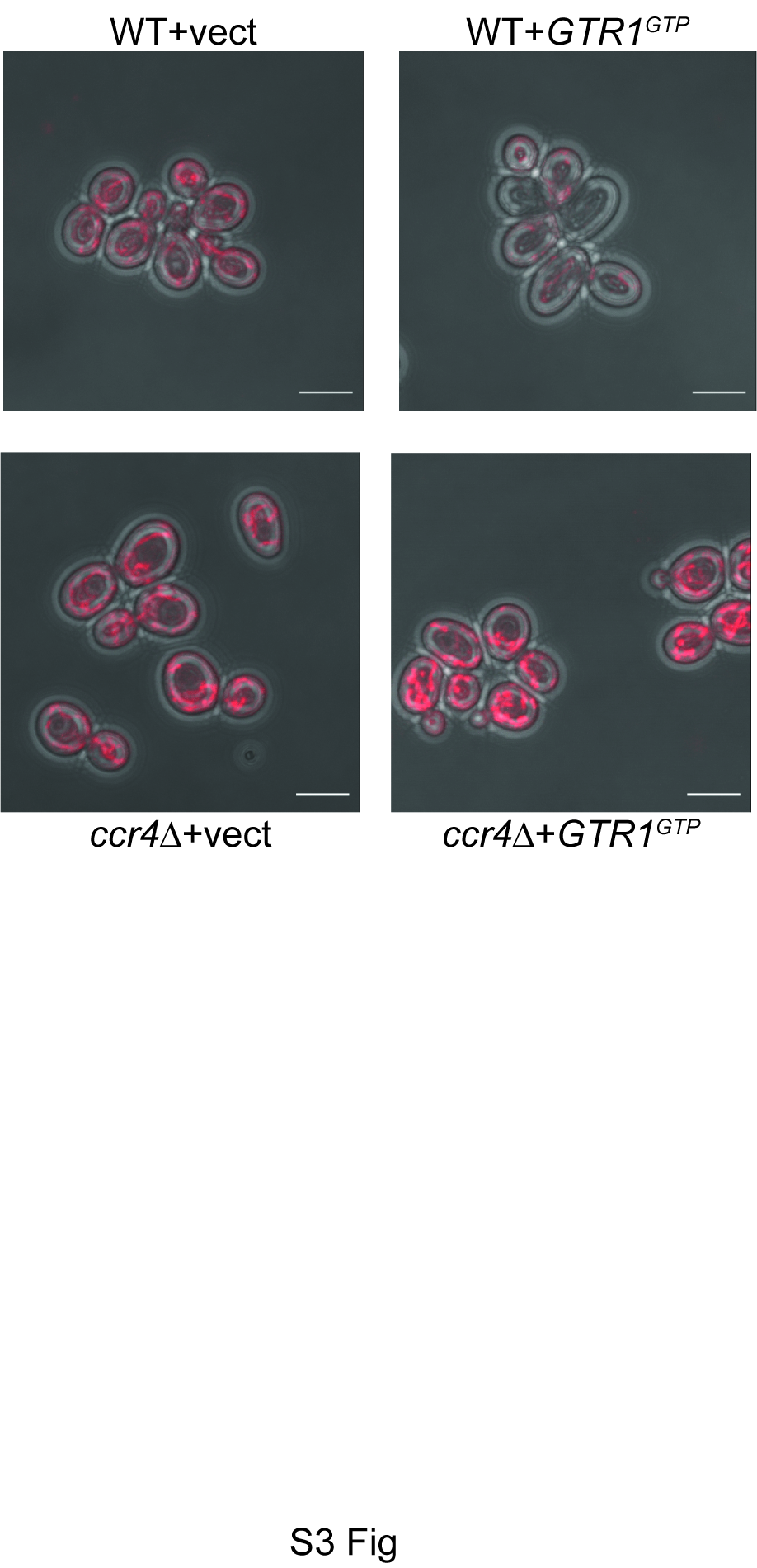

Supplement: S3 Fig — WT and ccr4Δ were transformed with control vector or vector expressing Gtr1GTP. Cells were grown to mid-log phase and then stained with Mitotracker, and then analyzed by confocal microscopy. Scale bar indicates 5 μm. (TIF) [file pgen.1009046.s003.tif]

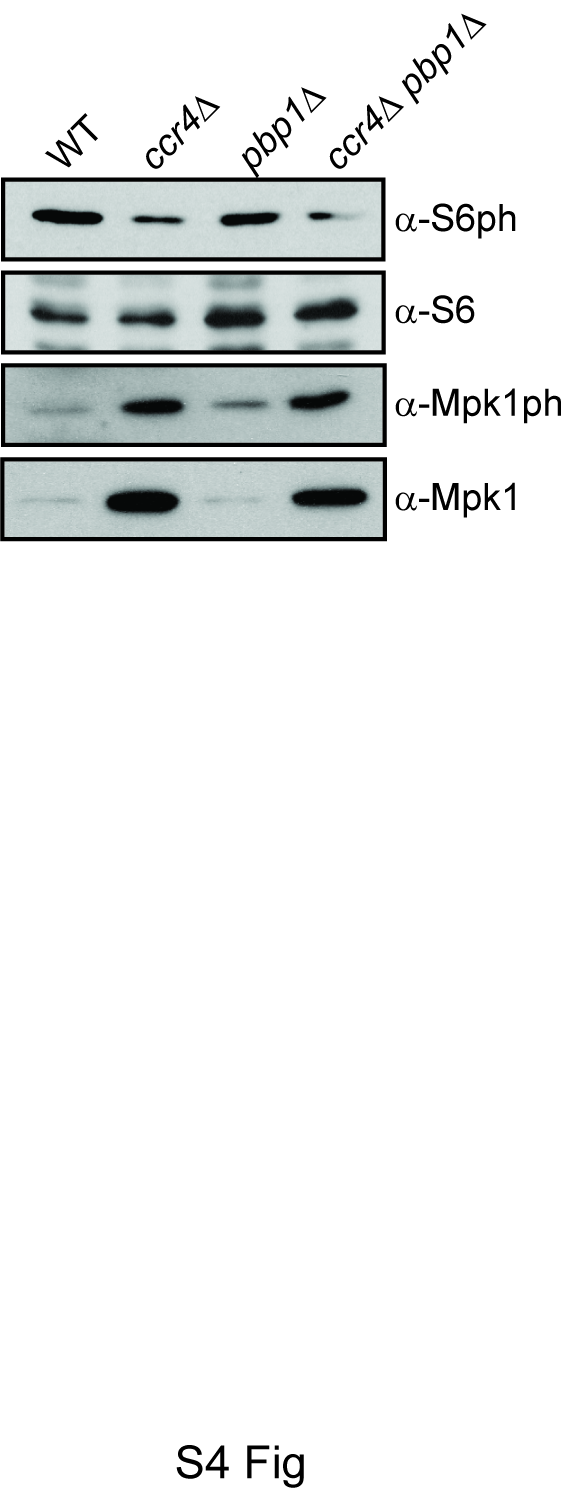

Supplement: S4 Fig — WT, ccr4Δ, pbp1Δ, and ccr4Δ pbp1Δ were analyzed for both TORC1 and Mpk1 signaling as indicated. Data are representative of three independent experiments. (TIF) [file pgen.1009046.s004.tif]
