# Supplementary material for: The Ccr4-Not complex regulates TORC1 signaling and mitochondrial metabolism by promoting vacuole V-ATPase activity
Source: PLoS Genet. 2020 Oct 16;16(10):e1009046. doi: 10.1371/journal.pgen.1009046 (PMC7592917; doi:10.1371/journal.pgen.1009046)
Supplement: S1 Table — (DOCX) [file pgen.1009046.s005.docx]

**S1 Table. Yeast strains.**

| **Strain** | **Description** | **Reference** |
| --- | --- | --- |
| BY4741 | *MATa his3-D1 leu2-D0 met15-D0 ura3-D0* | Dharmacon/  Open Biosystems |
| *ccr4Δ* | *MATa his3-D1 leu2-D0 met15-D0 ura3-D0 ccr4D::K KanMX* | Dharmacon/  Open Biosystems |
| *not4Δ* | *MATa his3-D1 leu2-D0 met15-D0 ura3-D0 not4D::K KanMX* | Dharmacon/  Open Biosystems |
| *caf40Δ* | *MATa his3-D1 leu2-D0 met15-D0 ura3-D0 caf40D::K KanMX* | Dharmacon/  Open Biosystems |
| *vph1Δ* | *MATa his3-D1 leu2-D0 met15-D0 ura3-D0 vph1D::K KanMX* | Dharmacon/  Open Biosystems |
| *vma3Δ* | *MATa his3-D1 leu2-D0 met15-D0 ura3-D0 vma3D::K KanMX* | Dharmacon/  Open Biosystems |
| *puf3Δ* | *MATa his3-D1 leu2-D0 met15-D0 ura3-D0 puf3D::K KanMX* | Dharmacon/ Open Biosystems |
| *puf4Δ* | *MATa his3-D1 leu2-D0 met15-D0 ura3-D0 puf4D::K KanMX* | Dharmacon/ Open Biosystems |
| *puf1Δ* | *MATa his3-D1 leu2-D0 met15-D0 ura3-D0 puf1D::K KanMX* | Dharmacon/ Open Biosystems |
| *tco89Δ* | *MATa his3-D1 leu2-D0 met15-D0 ura3-D0 tco89D::K KanMX* | Dharmacon/  Open Biosystems |
| *rtg1Δ* | *MATa his3-D1 leu2-D0 met15-D0 ura3-D0 rtg1D::K KanMX* | Dharmacon/  Open Biosystems |
| *pbp1Δ* | *MATa his3-D1 leu2-D0 met15-D0 ura3-D0 pbp1D::K KanMX* | Dharmacon/  Open Biosystems |
| YNL416 | *MATa his3-D1 leu2-D0 met15-D0 ura3-D0 ccr4D::NatNT2* | This study |
| YNL483 | *MATa his3-D1 leu2-D0 met15-D0 ura3-D0 Kog1-6xHA::HphNT1 Lst8-9xMYC:NatNT2* | This study |
| YNL815 | *MATa his3-D1 leu2-D0 met15-D0 ura3-D0 ccr4D::NatNT2 pbp1D::KanMX* | This study |
| YNL822 | *MATa his3-D1 leu2-D0 met15-D0 ura3-D0 ccr4D::NatNT2 rtg1D::KanMX* | This study |
| YNL831 | *MATa his3-D1 leu2-D0 met15-D0 ura3-D0 VMA1-EGFP::KanMX4* | This study |
| YNL833 | *MATa his3-D1 leu2-D0 met15-D0 ura3-D0 VMA1-EGFP::KanMX4 ccr4D::NatNT2* | This study |
| YNL835 | *MATa his3-D1 leu2-D0 met15-D0 ura3-D0 KOG1-6XHA::HphNT1 ccr4D::K KanMX LST8-9XMYC::NatNT2* | This study |
| YNL815 | *MATa his3-D1 leu2-D0 met15-D0 ura3-D0 ccr4D::NatNT2 pbp1D::KanMX* | This study |
| YNL821 | *MATa his3-D1 leu2-D0 met15-D0 ura3-D0 ccr4D::NatNT2 rtg1::KANMX* | This study |
| YNL846 | *MATa his3-D1 leu2-D0 met15-D0 ura3-D0 SNF1-6XHA::HphNT1* | This study |
| YNL850 | *MATa his3-D1 leu2-D0 met15-D0 ura3-D0 SNF1-6XHA::HphNT1 ccr4D::NatNT2* | This study |
| YNL885 | *MATa his3-D1 leu2-D0 met15-D0 ura3-D0 ccr4D::NatNT2 tor1D::KanMX* | This study |
| YNL886 | *MATa his3-D1 leu2-D0 met15-D0 ura3-D0 ccr4D::NatNT2 mpk1D::KanMX* | This study |
