# Supplementary material for: The Ccr4-Not complex regulates TORC1 signaling and mitochondrial metabolism by promoting vacuole V-ATPase activity
Source: PLoS Genet. 2020 Oct 16;16(10):e1009046. doi: 10.1371/journal.pgen.1009046 (PMC7592917; doi:10.1371/journal.pgen.1009046)
Supplement: S2 Table — (DOCX) [file pgen.1009046.s006.docx]

**S2 Table. Yeast plasmids.**

| **Plasmid** | **Description** | **Reference** |
| --- | --- | --- |
| p416ADH | *AmpR CEN6/ARSH4 URA3 ADH1prom; CYC1term* | (Mumberg et al., 1995) |
| p415ADH | *AmpR CEN6/ARSH4 LEU2 ADH1prom; CYC1term* | (Mumberg et al., 1995) |
| pADHLEU-CCR4 | *AmpR CEN6/ARSH4 LEU2 ADH1prom-CCR4-FLAG; CYC1term* | This study |
| pADHLEU-ccr4-1 | *AmpR CEN6/ARSH4 LEU2 ADH1prom-ccr4-1-FLAG; CYC1term* | This study |
| pADH GTR1^GTP^ | *AmpR CEN6/ARSH4 LEU2 ADH1prom-GTR1^Q65L^; CYC1term* | This study |
| pRS425 | *AmpR 2µ LEU2* | (Christianson et al., 1992) |
| pRS426 | *AmpR 2µ URA3* | (Christianson et al., 1992) |
| pSOD1 | *AmpR 2µ LEU2 SOD1* | (Chen et al., 2016) |
| pRS25 VMA6 | *AmpR 2µ LEU2 VMA6* | This study |
| pRS426 VMA13 | *AmpR 2µ URA3 VMA13* | This study |

**References**

Chen, H., Workman, J.J., Strahl, B.D., and Laribee, R.N. (2016). Histone H3 and TORC1 prevent organelle dysfunction and cell death by promoting nuclear retention of HMGB proteins. Epigenetics Chromatin *9*, 34.

Christianson, T.W., Sikorski, R.S., Dante, M., Shero, J.H., and Hieter, P. (1992). Multifunctional yeast high-copy-number shuttle vectors. Gene *110*, 119-122.

Mumberg, D., Muller, R., and Funk, M. (1995). Yeast vectors for the controlled expression of heterologous proteins in different genetic backgrounds. Gene *156*, 119-122.
