# Supplementary material for: The Ccr4-Not complex regulates TORC1 signaling and mitochondrial metabolism by promoting vacuole V-ATPase activity
Source: PLoS Genet. 2020 Oct 16;16(10):e1009046. doi: 10.1371/journal.pgen.1009046 (PMC7592917; doi:10.1371/journal.pgen.1009046)
Supplement: S3 Table — (DOCX) [file pgen.1009046.s007.docx]

**S3 Table. PCR primers.**

| **Primer** | **Sequence** | **Reference** |
| --- | --- | --- |
| Kog1 S3 | CTATTGTAAATATCTACAAGTGTGAAGACGAGAGAATTGATTATTTTCGTACGCTGCAGGTCGAC | This study |
| Kog1 S2 | CTCTTTTGCAGCTAAATGAAAGAAAAAAAAAGAAATGGCACATATCAATCGATGAATTCGAGCTCG | This study |
| Kog1midORFfor | ATGATACGTCGCTGGAGAGC | This study |
| Lst8 S2 | GTCACTTCGGTCCTTCAAGCTTGGTAGTTGATAAATTGT AAATTAATCGATGAATTCGAGCTCG | This study |
| Lst8 S3 | CAGTACGGTGGACACCACAAGGGAGCTGTATGTGTC GCATTAAACGATGTACGTACGCTGCAGGTCGAC | This study |
| Lst8midORFfor | ATTTGGCAACATGTTCAGCG | This study |
| GTR1 cloneFor | ATCACGTCTAGAATGTCGTCAAATAATAGGAAGAAAC | This study |
| GTR1 cloneRev | ATCACGCTCGAGTCAAGCGTAGTCTGGGACGTCGTATGGGTATTGGAAAAACTCTTTGGC | This study |
| VMA6clone For | ATCACGAAGCTTGAATTGTAGGAAGATGAAAAAGTG | This study |
| VMA6clone Rev | ATCACGCTCGAGATGGATCTGATTCTTAAAAAAAAATG | This study |
| VMA13clone For | ATCACGAAGCTTCATAAAATAAGAGGCTGCGACAGTC | This study |
| VMA13clone Rev | ATCACGCTCGAGCTTTTCTTTTCTATGGATGTTCTTC | This study |
| SOD1 for300 | ATCACGCTCGAGTGCGACTCACCCACTCAG | This study |
| SOD1 rev100 | ATCACGGGATCCCAAGGACATAAATCTAAG | This study |
| qTOR1 for | TGGAGTGGTGATTGGCAGTA | This study |
| qTOR1 rev | ACAGCCCCTACCCTAGTGCT | This study |
| qLST8 for | TTCAGCATTCCGACTCACAG | This study |
| qLST8 rev | CGCTAGAGGTCACCATCCAT | This study |
| qKOG1 for | CCTGATTGGGGTGTTAATGG | This study |
| qKOG1 rev | GCTTTTTGTTCATCGGTGGT | This study |
| qTCO89 for | TAGCTTCGCCCAGTTCCTTA | This study |
| qTCO89 rev | CCCTTTTCGCATCAATGTTT | This study |
| qYPK3 for | CTGAATACTGTGCGCCTGAA | This study |
| qYPK3 rev | CATTCCACCTTTTGGCAGTT | This study |
| qARO9midFor | GTCCCAGGGTATTGATGTCG | This study |
| qARO9midRev | GTCTTTGGGCCAACTGGTAA | This study |
| qFKS2mid For | CGTTCAAATGTTCGGTGGTA | This study |
| qFKS2mid Rev | TGCAAGTCCGGATAAGCTCT | This study |
| qPLB2midFor | CTGGCTCCGCTTTGACTT | This study |
| qPLB2midRev | TCAAATGGAGTGAACTCGAAAA | This study |
| qTPO1midFor | TTTCATATGTAGATTCTTTGGTGGTT | This study |
| qTPO1midRev | CCCATGACAGGCGATAAAAT | This study |
| qCIT2end For | TGGCCAAATGTAGATGCTCA | This study |
| qCIT2end Rev | AGGACTTTGGCCTTTCAATG | This study |
| qmCOBfor | GATTCATATGGATAGCGTAAGTCAA | This study |
| qmCOBrev | TGGAAATAATTGGGCTTACCA | This study |
| qmATP9for | TTGGAGCAGGTATCTCAACAAT | This study |
| qmATP9rev | GCTTCTGATAAGGCGAAACC | This study |
| qmCOX1for | AAGAAAGAGCTAAAGGCCCACT | This study |
| qmCOX1rev | TGCTGGTAGTTCAGTTGCACA | This study |
| qHXK1 for | TGGGTGAATTGTTGCGTCTA | This study |
| qHXK1 rev | CTGGCAGAGTGGTCTTGACA | This study |
| qHXK2 for | TGGAAAAGGGTTTGTCCAAG | This study |
| qHXK2 rev | TGGTGTCAAAGGTACGGTCA | This study |
